# Mapping MAVE data for use in human genomics applications

**DOI:** 10.1186/s13059-025-03647-x

**Published:** 2025-06-25

**Authors:** Jeremy A. Arbesfeld, Estelle Y. Da, James S. Stevenson, Kori Kuzma, Anika Paul, Tierra Farris, Benjamin J. Capodanno, Sally B. Grindstaff, Kevin Riehle, Nuno Saraiva-Agostinho, Jordan F. Safer, Jonathan Casper, Maximilian Haeussler, Aleksandar Milosavljevic, Julia Foreman, Helen V. Firth, Sarah E. Hunt, Sumaiya Iqbal, Melissa S. Cline, Alan F. Rubin, Alex H. Wagner

**Affiliations:** 1 The Steve and Cindy Rasmussen Institute for Genomic Medicine, Nationwide Children’s Hospital, Columbus, OH USA; 2 Bioinformatics Division, The Walter and Eliza Hall Institute of Medical Research, 1G Royal Parade, Parkville, Australia; 3 Department of Molecular and Human Genetics, Baylor College of Medicine, Houston, TX USA; 4 Brotman Baty Institute for Precision Medicine, Seattle, WA USA; 5 European Molecular Biology Laboratory, European Bioinformatics Institute, Wellcome Genome Campus, Hinxton, Cambridge, CB10 1SD UK; 6 The Center for the Development of Therapeutics, The Broad Institute of MIT and Harvard, Cambridge, MA USA; 7 UC Santa Cruz Genomics Institute, Santa Cruz, CA USA; 8 East Anglian Medical Genetics Service, Cambridge University Hospitals NHS Foundation Trust, Cambridge Biomedical Campus, Cambridge, CB2 0QQ UK; 9 Department of Medical Biology, University of Melbourne, Parkville, Australia; 10 Departments of Pediatrics and Biomedical Informatics, The Ohio State University, Columbus, OH USA

**Keywords:** Functional assay, Genomics, Genomic medicine, Multiplexed assays of variant effect, Variation representation specification, Deep mutational scanning, Massively parallel reporter assays, Global Alliance for Genomics and Health

## Abstract

**Background:**

Experimental data from functional assays have a critical role in interpreting the impact of genetic variants. Assay data must be unambiguously mapped to a reference genome to make it accessible, but it is often reported relative to assay-specific sequences, complicating downstream use and integration of variant data across resources. To make multiplexed assays of variant effect (MAVE) data more broadly available to the research and clinical communities, the Atlas of Variant Effects Alliance mapped MAVE data from the MaveDB community database to human reference sequences, creating an extensive set of machine-readable homology mappings that are incorporated into widely used human genomics applications.

**Results:**

Here, we map approximately 9.0 million individual protein and nucleotide variants in MaveDB to the human genome, describing the examined variants with respect to human reference sequences while preserving the data provenance of the original MAVE sequences. We then disseminate the results to major genomic resources including the Genomics 2 Proteins Portal, UCSC Genome Browser, Ensembl Variant Effect Predictor, and DECIPHER platform. Within these applications, MAVE variants can now be visualized and integrated with other relevant clinical and biological data, making additional knowledge available when performing variant interpretation and conducting other research activities.

**Conclusions:**

Mapping MAVE variants to human reference sequences and sharing the mapped dataset with several key human genomics applications enables a new and diverse set of applications for MAVE data. This study provides increased access to functional data that can assist in clinical variant interpretation pipelines and enable biomedical research and discovery.

**Supplementary Information:**

The online version contains supplementary material available at 10.1186/s13059-025-03647-x.

## Background

The use of high-throughput sequencing technologies in the clinical setting continues to grow, but shortfalls in available genomic evidence are contributing to a growing interpretation gap, where many more variants are being observed than can be classified as pathogenic or benign. Roughly half [1] of curated variants in the ClinVar database are classified as “variants of uncertain significance” (VUS) due to insufficient evidence supporting or refuting pathogenicity [2]. While in silico prediction tools exist and have improved substantially in recent years, they are not a replacement for experimental functional data according to clinical best practice [3–5]. Multiplexed assays of variant effect (MAVEs) can provide functional evidence to support variant classification by measuring the effects of thousands of variants in parallel [6, 7]. Commonly used MAVE designs include deep mutational scanning, which measures the functional effects of protein variants [8, 9], and massively parallel reporter assays (MPRAs), which interrogate regulatory elements like promoters and enhancers [10, 11]. As MAVEs produce functional scores for many variants chosen systematically, typically all single nucleotide or single amino acid changes in a given target, they are able to generate functional evidence for VUS before they are detected in a clinical context, providing evidence that can ultimately assist in clinical variant interpretation [12, 13]. MAVE data have already been incorporated into some ClinGen Expert Panel ACMG/AMP variant interpretation guidelines, e.g., for variants in *TP53* associated with Li-Fraumeni syndrome [14].

The increased use of MAVE experimental methods created a need for central repositories designed for MAVE experimental data and associated metadata. In 2019, MaveDB [15, 16] became the first such publicly accessible resource. With nearly 2000 submitted experimental datasets in MaveDB at the time of writing and more submitted every month, there is clear value in enabling the representation and exchange of these data, as well as improved guidance for how these data may be used to support the clinical classification of genomic variants. The Atlas of Variant Effects Alliance [17] is a consortium working to realize these goals and enable MAVE data generation and applications more broadly.

**Fig. 1:**
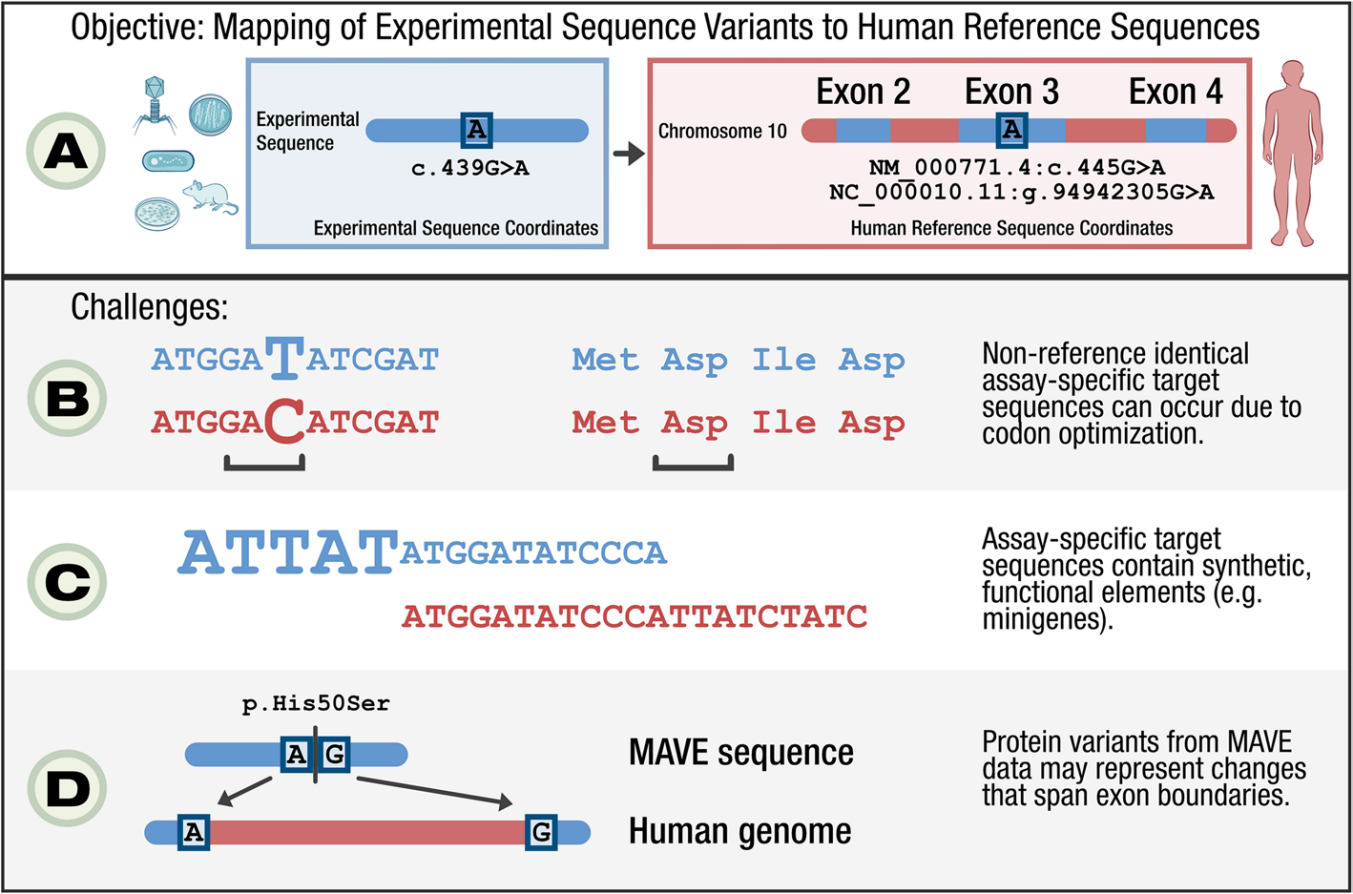
Objectives and challenges for mapping MAVE data to human reference sequences. **A** Data from MaveDB are described on user-submitted experimental sequences. To make these data accessible on human reference sequences, mappings are required to translate the experimental variant coordinates to human reference systems such as GRCh38. **B** MAVE sequences are often not identical due to structural features of the assay, such as codon optimization in polysome profiling assays [20]. In this example, there is a synonymous nucleotide difference between the target and reference sequences that optimizes translation of the sequence in the assay. **C** MAVE sequences can contain assay-specific functional elements that do not align to the human genome, such as minigenes used in saturation mutagenesis-based assays [21]. **D** MAVE protein variants may represent changes that would span exon boundaries on the human genome, but occur on a contiguous region on reverse-transcribed assay sequences [22, 23]

For most entries, MaveDB describes sequence changes with respect to a *target sequence* uploaded by the submitter. However, as the target sequence is not necessarily identical to a human reference sequence or associated with a commonly used accession number (e.g., Ensembl/GENCODE [18], RefSeq [18, 19], or GRC genome assemblies), a challenge emerges concerning the standardized representation of variation (Fig. 1). While the target sequence is necessary for the precise description of the experiment, this design presents challenges to interoperability between MAVE datasets and variants described on human reference sequences, including those in major knowledge bases or reported by clinical sequencing pipelines.

To address this challenge, we generated a MAVE dataset mapping for the FAIR [24] and computable exchange of variation data using open-source tools and databases [25–28]. In addition to representing MAVE variants on human reference sequences, our dataset mapping preserves the original MAVE sequence context, maintaining data provenance and ensuring that experiment-specific sequence differences are presented to downstream users. We integrated these mapped data into several common tools used for human genomics research and clinical variant curation, including the Genomics 2 Proteins Portal (G2P) [29], UCSC Genome Browser [30], the Ensembl Variant Effect Predictor (VEP) [31], the DECIPHER platform [32], the ClinGen Data Platform [33], and Shariant [34]. Through these efforts, we have also developed a reproducible workflow that can be applied to mapping future score sets in MaveDB. The dataset generated from our mapping approach closes an important gap for the application of MAVE data in genomic medicine and human health research.

## Results

### Mapping MaveDB variants to human reference sequences

MaveDB datasets based on human target sequences were selected for generating a set of variant mappings. From the most recent MaveDB release [35], 1064 MAVE score sets were identified as targeting human sequences, together totaling more than 9 million individual variants (variants describing multiple sequence changes were mapped and counted separately) and providing a large and heterogeneous dataset upon which variant mapping could be performed. Of the 1064 selected score sets, 1023 described protein coding genes while the remaining 41 covered regulatory and other noncoding elements. Among the 1064 examined score sets, 582 target sequences were specified at the amino acid level while the remaining 482 were specified at the nucleotide (DNA) level (Additional file 1: Fig. S1).

After extracting the relevant score set metadata, we then generated a set of variant mappings across the score sets to enable dissemination to downstream implementers. This variant mapping procedure was accomplished in three sequential steps (see Methods and Additional file 2). First, as homologous sequence annotations were not universally available across MAVE experiments, we used the BLAST-like Alignment Tool (BLAT) [27] to align MaveDB target sequences to the GRCh38 human genome assembly. Second, we analyzed this initial alignment data to computationally infer compatible RefSeq transcripts associated with the target sequences (Fig. 1A, D). Third, we processed the MAVE variants using the Global Alliance for Genomics and Health (GA4GH) Variation Representation Specification (VRS) and combined the resulting variant objects with the associated score set metadata to create the resultant mapped dataset (Additional file 1: Fig. S2).

For each MaveDB score set, the mapped dataset contains a list of MAVE variants described as pairs of “pre-mapped” and “post-mapped” variant objects. The pre-mapped form describes each variant with respect to the MAVE target sequence while the post-mapped form describes each variant with respect to the corresponding human reference sequence. Additionally, a unique, computable digest was assigned to identify each pre-mapped and post-mapped variant. Assigning variant identifiers maintains data provenance, ensuring that the original MAVE sequence context was preserved in the mapping. This is particularly important when applying MAVE evidence in clinical variant assessment, as an understanding of the experimental context in which the MAVE data was generated is essential in ensuring that functional evidence is appropriately applied.

Of the 1064 human score sets that were available for analysis, 1057 were ultimately processed using our variant mapping procedure. Of the seven score sets that failed to map, there were six score sets where a RefSeq protein identifier was unable to be selected given the alignments. The other score set was unable to be aligned using its target sequence. Across the 1057 processed score sets, the average number of examined functional measurements per score set was 2833 measurements, with a median of 1289 measurements (Additional file 1: Fig. S3).

For the 1057 processed human score sets in MaveDB, concordant mappings were observed for 68.44% (6,158,451/8,998,024) of examined MAVE variants, where concordance is defined as equivalence in the reference allele sequences of each pre-mapped and post-mapped variant pair. The remaining 31.56% (2,839,573/8,998,024) of discordant variant pairs were caused by factors including (1) protein changes in the MAVE score set mapping across exon boundaries, (2) non-homologous sequence content from MAVE target sequences preventing a reference match, and (3) supplied variants occurring past the original target sequence length. Discordant variants were not distributed uniformly across the MaveDB score sets, with 736 score sets comprising 3,031,995 variant pairs containing no discordance.

The overwhelming majority of variant mapping discordance in our dataset can be attributed to non-homologous content in the MAVE sequences. Specifically, 98.84% (2,806,609/2,839,573) of discordant pairs were found in 261 score sets from a single study [36] that used Protein Data Bank [37] sequences to produce biophysical models for assessing protein folding stability. The target sequences in these score sets were heavily mutagenized, interspersed with point mutations that led to a substantive divergence from the relevant human reference sequences. As a result of this divergence, we were often unable to generate alignment data for the entire target sequence. Furthermore, in the regions that did successfully align, there was a greater tendency for the MAVE sequence to differ from the human reference sequence at their respective pre-mapped and post-mapped variant positions.

Across the 6,158,451 concordant variant pairs in our dataset, we identified 1,048,823 unique pre-mapped variants that mapped to 1,018,091 unique post-mapped variants. The reduction from ~ 6.1 million to ~ 1.0 million unique variants is due to multiple experimental measurements for some pre-mapped variants across several score sets. Across all evaluated score sets, 899 had target sequences that differed from the human reference sequence, resulting in 820,427 (80.6%) unique post-mapped variants that were enabled through our mapping approach. The remaining 158 score sets were described on MAVE target sequences that were identical to their respective human reference sequences, and 197,664 (19.4%) unique post-mapped variants had an equivalent pre-mapped representation.

### Integrating mapped MaveDB data into genomic databases and tools

The immediate effect of our work to map MAVE variants to human references was to enable the integration of the mapped score sets into widely used human genomics resources. These integrations enable MAVE data to be used in a multitude of downstream clinical and research applications. The result of our efforts is availability of subsets of these mapped data across the following major community resources.

#### MaveDB

While the MaveDB resource was the source of the pre-mapped variants in this study, as a result of our work, all post-mapped variant records have also been added to the MaveDB database for programmatic access through an API and dataset-specific download files. The MaveDB REST API [38] provides pre- and post-mapped VRS objects for all variants mapped as part of a queried study through the/mapped-variants endpoint, and responses are returned as JavaScript Object Notation (JSON) formatted documents. Files containing the mappings for each score set are also retrievable via the MaveDB web interface (Additional file 1: Fig. S4). Future data releases on the MaveDB platform will use our mapping library to perform automatic mapping of newly added datasets, enabling this feature for any dataset on the platform.

#### Genomics 2 Proteins Portal

**Fig. 2:**
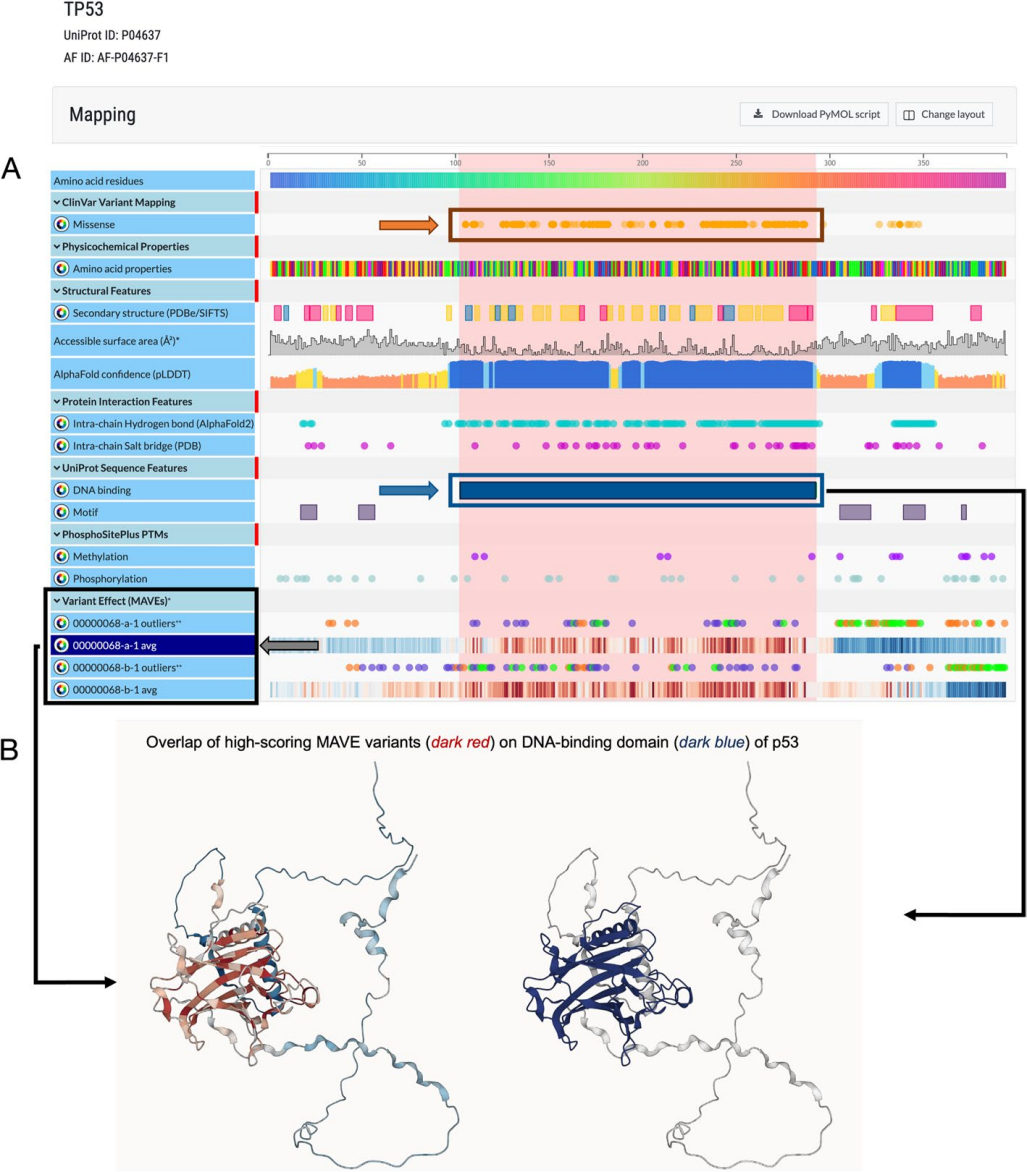
Integration and visualization of MAVE data into the Genomics 2 Proteins (G2P) Portal. The G2P Portal displays the MAVE scores (average score per residue) and outliers (mutations with MAVE scores that are 99th percentile top and bottom of the distribution of the corresponding score set) on both protein sequences and three-dimensional structures. **A** MAVE data mapped on the protein sequence along with clinical data and additional sequence and structure annotations such as protein secondary structures, protein–protein interactions, and domain annotations. For the selected gene *TP53* [43] (https://g2p.broadinstitute.org/gene/TP53/protein/P04637), the mapping showed an overlap across the locations of pathogenic mutations in ClinVar (indicated using orange rectangle and arrow), the DNA-binding domain annotation from UniProt database (indicated using dark blue rectangle and arrow), and the hotspot according to average MAVEs (indicated using gray rectangle and arrow). The presence of high scoring MAVE variants indicates a potential effect on the DNA-binding domain for *TP53*. **B** MAVE data mapped on the AlphaFold-predicted protein structure, highlighting the hotspot identified in MAVE score set urn:mavedb:00000068-a-1 (indicated using a black arrow) on the DNA-binding domain (dark blue) of the tumor suppressor protein p53

Genomics 2 Proteins (G2P) Portal [29] is an online discovery platform for linking genomic data to protein sequences and structures. The portal provides a user interface for exploring genetic variations, readouts from genetic perturbation assays, and protein features on the protein sequence and structure at the amino acid residue level to help interpret the molecular effect of variations. The portal integrates data from large genomic (gnomAD [39], ClinVar [2], and HGMD [40]) and proteomic databases (including UniProt [41], PDB [37], and AlphaFold [42]) as well as enabling users to perform customized mapping of genetic variations to proteins (Fig. 2).

We integrated 706 score sets describing MAVEs for 456 unique human protein coding genes into the G2P Portal. In addition to single amino acid residue substitutions (“point mutations”), MAVEs are available for pairwise residue mutations (“pairwise mutations”) for 9 out of 456 genes (Additional file 1: Fig. S5a and Additional file 3). MAVE data were mappable for > 90% of the residues of 38 proteins based on the length of the canonical protein isoform from UniProt (Additional file 1: Fig. S5b).

All MAVEs for both point and pairwise mutations for each gene and score set are displayed in the G2P Portal as heatmaps (Additional file 1: Fig. S5c, d) and are downloadable as JSON files. Additionally, for each gene and score set pair, mutations with top and bottom 99th percentile of MAVEs are displayed in the context of protein sequence annotations of structural (secondary structure, residues’ solvent accessibility, etc.) and functional features (e.g., domain, active sites) (Additional file 1: Fig. S5e). For example, MAVE readouts for *TP53* and score set urn:mavedb:00000068-b-1 range from − 5.39 to 2.80. Mutations with scores greater than 1.92 (top 99th percentile) and less than − 2.61 (bottom 99th percentile) were annotated in the “Protein sequence annotation” viewer of the portal. The filtering was performed for a clear visualization. These top and bottom 99th percentiles of MAVEs can also be mapped on their corresponding protein structure positions and are downloadable in tabular format from the portal. A list of genes with MAVE data in the G2P Portal can be viewed under the “Protein Features” section on the portal’s statistics page [44]. We also used the mappings to calculate average MAVE scores for each coding reference amino acid position for display alongside protein sequences and structures. The integration of MAVEs with protein sequence and structural features facilitates interpreting MAVE data on clinically relevant genes such as *TP53* [45] (Fig. 2).

#### UCSC Genome Browser

**Fig. 3:**
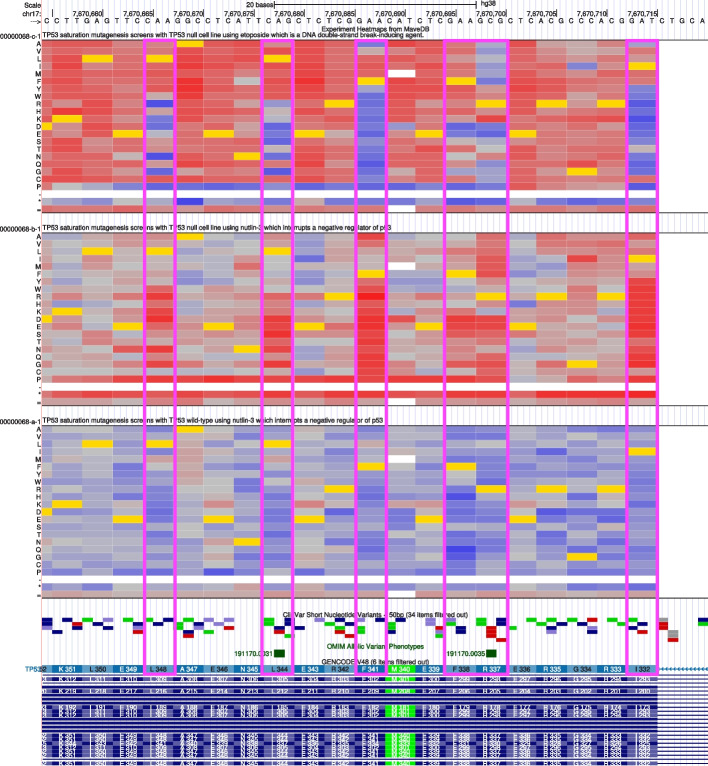
Integrating MAVE data as a custom track hub in the UCSC Genome Browser. An illustration of MAVE data in the UCSC Genome Browser. MAVE protein variant positions are mapped to their corresponding genomic coordinates, and consequence scores are reported for each variant via mouseover text. This example illustrates the MaveDB score sets urn:mavedb:00000068-a-1, urn:mavedb:00000068-b-1, and urn:mavedb:00000068-c-1. In these experiments, mutated *TP53* was added to a cell line depleted of wild-type *TP53* with the following treatments: etoposide, a DNA double-strand break-inducing agent (top); nutlin-3, which impairs proliferation of *TP53* by suppressing the interaction between p53 and MDM2 (middle); and nultlin-3 plus wild-type *TP53* (bottom) [45]. The heatmaps render the log ratio of cell counts before and after treatment, with colors ranging from blue (lowest) to red (highest). Amino acid positions 332, 337, 338, 341, 344, and 348 (indicated by pink boxes) show contrasting responses in the non-conservative amino acid substitutions (roughly, the lower two-thirds of each heatmap). These positions are involved in the formation of p53 tetramers. Other data informing the clinical impact of variation in these positions is illustrated by the pathogenic variants (red) in the ClinVar SNVs, and the OMIM [49] allelic variant phenotypes 191170.0031 (Li-Fraumeni syndrome) and 191170.0035 (adrenocortical carcinoma). These data are accessible via a UCSC Genome Browser session [50] (https://genome.ucsc.edu/s/mcline/MaveDB_TP53_Figure)

The UCSC Genome Browser [30] is a widely used and highly customizable web platform supporting genome research and includes annotations from many datasets (referred to as “tracks” on the platform) of clinical and research relevance. Its power lies in allowing users to visualize these annotations in a genomic context together with other tracks of annotation data. This enables users to recognize relevant trends, such as putative connections between annotation data values and types of genomic regions, which can then be tested quantitatively with the built-in Table Browser [46] tool. We have created a genome browser track hub of these mappings, which displays each protein variant in a genomic context with the associated scores. The track hub renders these scores as a heatmap, in which each column represents the mapped genomic location of the variants scored, each row represents an alternate allele, and cells are colored on the blue/red color spectrum in proportion to the score (Fig. 3). This allows rapid identification of the nucleotides where SNVs tend to incur greater loss of function, suggesting nucleotide positions which are more critical to protein function and/or RNA stability; these positions can then be compared to the cross-species genomic conservation scores available in the browser’s Conservation track group. The MaveDB Genome Browser track hub can be accessed as a custom track hub [47] and under the UCSC Genome Browser Public Session gallery [48], and the data have also been incorporated into a native track at UCSC for ease of access. Each score set in MaveDB with mapped variants also includes a link to the associated mappings track in the UCSC Genome Browser for convenient navigation between the two resources.

#### Ensembl VEP

**Fig. 4:**
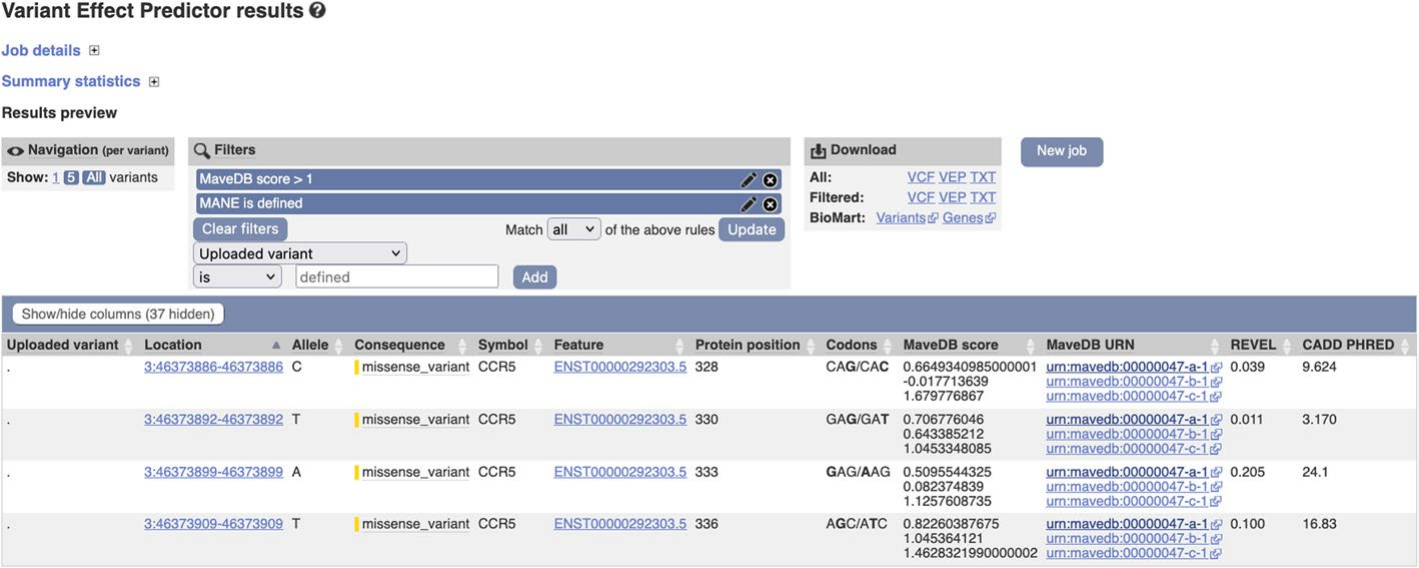
MAVE data available within the Ensembl Variant Effect Predictor. Ensembl VEP [52] (https://www.ensembl.org/Multi/Tools/VEP) output showing a batch of variants (see Additional file 4) annotated with MAVE results. The scores and a link to the associated score set information in MaveDB are reported, when available. Results can be filtered for the specific MaveDB score ranges considered to be of interest. The displayed example includes a subset of MAVE variants in the *CCR5* gene across experiment set urn:mavedb:00000047 along with their associated REVEL [53] and CADD PHRED [54] scores

Ensembl VEP [51] is an open-source tool for the annotation and prioritization of genomic variants. It predicts variant molecular consequence and mines aggregated publicly available knowledge to report extensive information about a variant loci. Available information includes highly informative, but relatively sparse variant phenotype associations and comprehensive, but less reliable predictions of pathogenicity. MaveDB results complement these data providing comprehensive informative results over many genomic regions. Ensembl VEP has three interfaces which have been designed to suit different use cases: (1) a highly configurable command line tool which can be used as the basis for large-scale variant annotation and filtering pipelines, (2) a REST API which enables on-the-fly annotation for use in in web displays, and (3) a simple web interface which facilitates the analysis of batches of up to 2 million variants. By leveraging the genomic mapping of MaveDB data, we were able to integrate MAVE score sets into the three respective interfaces within Ensembl VEP (Fig. 4 and Additional file 4). This integration streamlines community access to these data and allows convenient integration into large-scale variant annotation pipelines.

##### DECIPHER

**Fig. 5:**
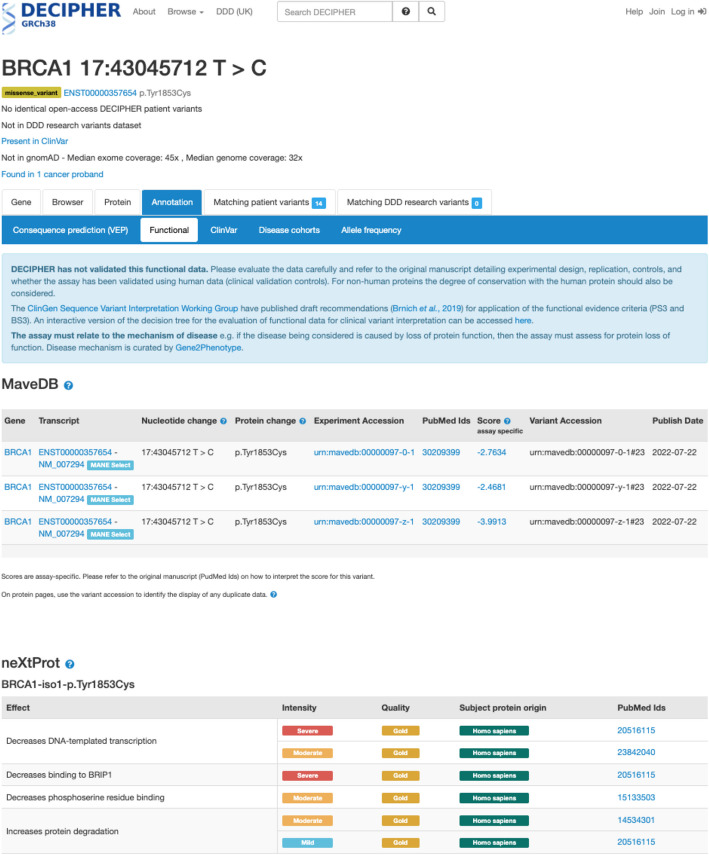
Integrating MAVE data into DECIPHER. The nucleotide change, protein change, experiment accession, PubMed ID, assay-specific variant effect score, variant accession, and publish date are included for MAVE data displayed in DECIPHER, with links to the experimental details and score set in MaveDB. DECIPHER also includes an interactive decision tree to assist in evaluating the functional data for clinical variant interpretation. The displayed example highlights the functional consequences of a tyrosine to cysteine substitution at residue 1853 in *BRCA1* [61] (https://www.deciphergenomics.org/sequence-variant/17–43045712-T-C/annotation/functional) across three different score sets. In this example, MAVE evidence can be linked with neXtProt annotations [62] to provide insights into the potential impact of the variant on biological function

DECIPHER [55] is a global resource that shares phenotype linked variant data from rare disease patients to support research and diagnosis, and provides variant interpretation interfaces [32, 56]. Displaying MAVE data in DECIPHER increases the discoverability of these data for clinicians, clinical scientists, clinical researchers, research scientists, and curators who use DECIPHER. MAVE data has already been incorporated into international guidelines for variant interpretation (e.g., for variants in *TP53* associated with Li-Fraumeni syndrome [14]) and also has enormous potential in assisting the re-classification variants of unknown significance (e.g., variants in *BRCA1* associated with cancer susceptibility [57, 58] and *MSH2*/*MLH1* associated with Lynch syndrome [59, 60]). We incorporated the mapped MAVE data into DECIPHER, allowing for this data to be displayed across DECIPHER’s user interfaces and enhancing data accessibility. Specifically, the MAVE data is displayed on functional data tabs which are accessed from DECIPHER patient records, in addition to variant pages and protein variant pages accessed through the site search tools (Fig. 5).

#### ClinGen Linked Data Hub

The ClinGen Linked Data Hub [63] (LDH) is a RESTful API service built on Linked Open Data principles [64] that aggregates excerpts of pertinent variant data from a variety of external sources. Through evidence aggregation, the LDH assists users in performing variant curation with the ClinGen Data Platform [33]. The LDH works in conjunction with the ClinGen Allele Registry [65] which is a canonical on-demand variant naming service. To incorporate MAVE data into the LDH, we submitted the mapped variants to the ClinGen Allele Registry and assigned Canonical Allele Identifiers (CAid) and Protein Allele Identifiers (PAid), enabling ingestion into the LDH. With the mapped MAVE data available in the LDH, users have access to functional evidence that can assist in variant curation efforts.

Users can access the MaveDB data via the LDH API by either using the LDH MaveDBMapping document’s entity ID (score set accession + “#” + variant number; e.g., urn:mavedb:00000001-a-1#1) or by searching for the associated variant CAid or PAid. Accessing the MaveDBMapping documents using the variant CAid or PAid allows users to easily access MaveDB data for the variant of interest from multiple MaveDB experiments or score sets simultaneously alongside pertinent data from other sources. The LDH API can also be used to return all MaveDBMapping documents from a particular score set, enabling bulk retrieval. Leveraging both ClinGen CAids/PAids and GA4GH VRS IDs allows for straightforward data aggregation of variants by identifier from groups that have adopted one or multiple data standards and provides the users with the level of specificity required for their application. MaveDBMapping objects can be queried through LDH API [66] and UI [63] endpoints (Additional file 1: Fig. S6).

#### Shariant

Shariant [34] is a controlled-access platform to allow inter-laboratory automated sharing of clinically curated variants and structured evidence across Australian and New Zealand laboratories. The platform is configured to consume CAids from the ClinGen Allele Registry, which will be used to accomplish the initial data exchange between MaveDB and Shariant. This underscores the importance of integrating and supporting data standards, as the submission of VRS objects to the ClinGen Linked Data Hub is an essential step for generating the CAids that Shariant requires. MaveDB data linked to CAids is being made available to Shariant users as part of a pilot focused on user testing and feedback. Access to Shariant is restricted to Australian and New Zealand laboratories conducting clinical-grade testing.

## Discussion

In this study, we mapped variants described in multiplexed assays of variant effect (MAVE) data to human sequence assemblies for use in clinical and research genomics applications. Leveraging the GA4GH Variation Representation Specification (VRS) [28] and associated open-source bioinformatics tools, we mapped over 9.0 million individual variants in MaveDB for downstream reuse. Our approach is informed by FAIR data principles and enables semantically precise representation of variants for data provenance.

While the vast majority of our data was mappable, this exercise also highlighted practical challenges in the mapping of experimental data to likely comparable changes in the human genome. For example, when a measured protein change from an experiment requires a multi-nucleotide change that spans an exon boundary, the corresponding genomic reference sequence coordinates can span thousands of nucleotides. Should such measurements map to multiple *in-cis* variants, or a single, very large variant that also covers the intronic space? In other cases, segments of a target sequence designed for specific experimental properties may not align to known human reference sequences, limiting our ability to interpret variants that are reported at those unaligned positions of the target sequence.

An important limitation of these data is identified by our study, through analysis of multiple score sets where the target and reference sequences resulted in alignments with highly discordant sequences. While our workflow can generate variant mappings when sequence divergence occurs, this observation highlights both the importance of maintaining the provenance of the functional evidence and the need for careful analysis when applying mapped MAVE data in downstream analyses. Researchers using these data are encouraged to assess the provenance of variant mappings to determine the degree of concordance between target and reference sequence, and draw experimental conclusions accordingly. Guidance on how to interpret alignment characteristics, experimental functional scores, and associated experimental metadata are areas of recommendation under consideration by the MAVE expert community, particularly within the Atlas of Variant Effects Alliance.

The responsible use of these data for clinical purposes in particular requires a high level of confidence that the MAVE assay relates to the mechanism of disease. For each specific gene-disease relationship in question, ensuring that the MAVE assay is a valid predictor of pathogenicity will be essential. This is likely to be especially challenging for genes with multiple disease associations and for proteins with multiple functional domains, and curated knowledge bases like the Gene Curation Coalition [67] and Gene2Phenotype [68] are helping to bridge this gap. We believe that the mapping of MAVE data to human reference sequence assemblies provides another crucial part of the foundation for the development of guidelines for the use of MAVE data, by providing common sequence assemblies for the evaluation of MAVE score sets and facilitating comparison between assay results and independently classified variants.

Our study also illustrates the disparate mechanisms by which variant knowledge is represented, integrated, and used by downstream resources. While GA4GH VRS provides a precise mechanism for addressing the complexity of representing variants across both experimental target and common reference sequences, its relatively recent emergence as a variant representation standard places it in a genomic data ecosystem alongside several established variant representation standards, including the Human Genome Variation Society (HGVS) nomenclature [69]. Recognizing this, we have used open-source translation tools [70] to annotate all mapped variants using HGVS for ingestion into downstream platforms that do not natively accept VRS objects. We were also required to consider methods for mapping protein variants to the genomic reference space for platforms that do not accept protein-level variant descriptions. These methods together have proved effective for integrating mapped MAVE data into several downstream resources, including the Genomics 2 Proteins Portal, UCSC Genome Browser, Ensembl VEP, the DECIPHER platform, and ClinGen Linked Data Hub resources, with additional integrations forthcoming.

Looking forward, we believe our approach could be extended to mapping MAVE variants from non-human score sets in MaveDB. MAVE data can investigate biologically meaningful processes across a range of other organisms including mice, yeast, bacteria, and plants, and data from human genes could have great utility for researchers studying variation in other organisms. By leveraging open-source resources such as SeqRepo and VRS-Python [71], we are able to register user-provided target sequences, normalize variants on these sequences, and assign unique identifiers to these objects, allowing for data provenance to be preserved. As our approach is organism-agnostic for describing pre-mapped variants, we can envision adapting our tooling to map these variants to non-human reference sequences, improving species coverage in MaveDB.

## Conclusions

The impact of genome and exome sequencing on human research and clinical practice is hindered by challenges in variant interpretation. Multiplexed assays of variant effect (MAVEs) provide a high-throughput functional assessment tool for variants in genes of relevance to human health and disease, and thousands of MAVEs have been developed and results submitted to the centralized MaveDB data repository. We created a substantial set of variant mappings across human score sets in MaveDB, allowing for the precise representation of MAVE data with respect to human reference sequences. This effort has enabled the integration of MAVE score sets into multiple variant annotation and evaluation platforms, ensuring that MAVE data is disseminated to the scientific community. We also discuss current challenges in the use of these data in human genomics applications and the need for expert communities like the Atlas of Variant Effects Alliance to address these remaining gaps. We believe the mapped data from this study will help advance those efforts, and the data integrations at the Genomics 2 Proteins Portal, the UCSC Genome Browser, Ensembl VEP, DECIPHER platform, ClinGen Linked Data Hub, and others will provide useful tools for advancing MAVE-informed genomic variant interpretation efforts.

## Methods

### Extraction of metadata from the MaveDB API score sets endpoint

When uploading score sets to MaveDB, one can choose to provide metadata that can assist downstream users in data interpretation. These metadata fields include the gene targeted by the MAVE experiment, the examined target sequence, and links to relevant sequence identifiers from databases such as UniProt [41]. As metadata availability can vary across score sets, it was important to analyze the structure of this information to ensure that it was appropriately processed. Accordingly, we investigated metadata formatting across our MaveDB subset, uncovering differences in structure that helped inform data processing steps in our variant mapping workflow (Additional file 5: Table S1).

During this analysis, six variables were extracted from the MaveDB score set metadata. These variables were target sequence (string of nucleotides or amino acids), target sequence type (DNA or protein), target (e.g., *CXCR4*), UniProt ID (if available), target type (e.g., protein coding), and the uniform resource name (URN) (e.g., urn:mavedb:00000048-a-1). These data elements were selected as they were the minimum information needed to determine the genomic coordinates targeted by an assay in a MaveDB score set.

### MAVE target sequence alignment and reference sequence selection

With these key data elements extracted, MaveDB target sequences were aligned to the human genome to select a set of representative genomic coordinates. This was achieved by providing each target sequence as input to the BLAT alignment tool, returning a list of possible genomic coordinates for the sequence. After running BLAT, a series of filtering and validation steps were performed to select the set of representative genomic coordinates for the target sequence. These specific steps are described in further detail in the supplementary materials [27, 41, 72–75] (see Additional file 2).

Following the alignment step, an additional procedure was performed to select a representative transcript sequence for MAVE variants that were represented on protein subsequences. Specifically, given the gene for a target sequence and its associated genomic coordinates, we followed community guidelines for selecting a canonical RefSeq [19] transcript, with an emphasis placed on selecting a Matched Annotation from NCBI and EMBL-EBI (MANE) [76] Select transcript as this is a standard for reporting clinical variants. An offset was also computed to determine the precise location of the MAVE sequence in the protein reference sequence. These steps leveraged the Biocommons SeqRepo [25] Python package/SQLite database, Universal Transcript Archive (UTA) [26] database, and GenomicMedLab Common Operations on Lots of Sequences (Cool-Seq-Tool) [77] Python package to select the representative RefSeq protein sequences and offsets. The precise transcript selection workflow is further described in the supplementary materials [19, 25, 26, 76, 77] (see Additional file 2).

### Creating variant mappings using GA4GH genomic knowledge standards

With the relevant human reference sequence data determined, MAVE variants were converted to VRS objects to build the variant mapping sets. First, each variant in a MAVE score set was converted to a VRS allele using its assigned positions, providing a computational representation of the assayed variant to be generated and using conventions best-suited to variant search and FAIR sharing. Specifically, the reported variants were translated into a VRS allele structure, a new sequence digest was computed and registered in SeqRepo, the allele was renormalized to full-justification [28], and the allele identifier was computed. In instances where multiple in-cis variants were described, this process was run separately for each component variant and a VRS CisPhasedBlock object was generated from the set of all in-cis variants. All processed pre-mapped and post-mapped variants were represented using VRS version 2.0.

The process described above was then repeated using the reference sequence information to create the mapped variants. For protein variants, the human reference sequence digest was determined while the offset was added to start and end position values. For genomic variants, the appropriate locations in the alignment were determined and replaced the previous start and end position values. After updating the mapped variant locations, a new allele digest was computed, allowing for the mapped variant to have a distinct identifier. When multiple variants were reported, new VRS alleles were created for each variant and combined in a VRS CisPhasedBlock.

In addition to storing the pre-mapped and post-mapped variants, appropriate metadata was added to each score set to assist users in downstream processing and analysis. The newly created pre-mapped and post-mapped objects were combined with the score set metadata files to generate the mapping files. Within the mapping files, we constructed a “computed_reference_sequence” attribute, storing the target sequence, sequence type, and sequence digest. We also created a “mapped_reference_sequence” attribute, storing the RefSeq accession, sequence type, and corresponding sequence digest. After this step, each variant mapping pair was annotated to include the reference sequence at the defined allele location. While this data is redundant and retrievable in downstream systems, its presence served as a useful concordance measure for each pre-mapped and post-mapped variant pair assessed during our study. Finally, an HGVS nomenclature description was added to each post-mapped variant to improve interoperability with downstream systems. Following the creation of the respective attributes, all processed score sets were saved as JSON files, compressed, and uploaded to a publicly accessible URL (see Availability of data and materials).

### Development of a reproducible process for mapping MaveDB variants

To support continual MaveDB mapping efforts, we released our mapping pipeline as a Python software package. The key phases of the mapping workflow were constructed as separate modules, and additional methods were included to manage data acquisition from external sources. An included command-line interface enables end-to-end execution of the mapping workflow for a requested MaveDB score set, producing a JSON file that combines the mapped variants and score set metadata together (see Additional file 6). The linking of the variant mappings and score set metadata provides key contextual information (e.g., the gene targeted by the score set) that can ultimately inform downstream integration efforts. The software was published to the Python Package Interface [78] (PyPI).

### Integrating MaveDB data in the Genomics 2 Proteins Portal

A separate pipeline was developed to efficiently access the MaveDB data, preprocess the score sets per gene, and perform further processing on the scores to map and visualize them on protein sequence and structure space using the G2P Portal [29]. The preprocessing step involved filtering out non-human genes, as the G2P Portal is a resource for analysis of human genes. Next, the MAVE scores were filtered for single nucleotide variants (SNVs) with protein coding consequences (missense, nonsense, and synonymous variants), as the G2P Portal is a human protein-coding genome-wide platform. All preprocessed data were then stored to the G2P Portal Google Cloud Storage for dynamic access and visualization. The score sets are updated regularly in the G2P Portal as part of a biannual data update plan or with a new MaveDB release.

The data were then integrated into the G2P Portal and visualized in three ways: (1) the entire mutagenesis data as heatmaps (Additional file 1: Fig. S5), (2) amino acid residue-wise scores on protein sequence, and (3) on protein three-dimensional structure (Fig. 2). For protein residue-wise score visualization, MAVEs were post-processed in two methods. Average readouts were computed at each amino acid position for a summary readout, and the top and bottom 99th percentile of scores were selected for visualization of outliers. During post-processing, only scores where the RefSeq transcript for the variant in the MAVE score set corresponded with the canonical protein isoform (determined via the G2P3D API providing the Gene-Transcript-Protein Isoform-Structure identifier mapping [79]) were mapped at the target protein’s sequence and structure.

### Integrating MaveDB data into the UCSC Genome Browser

Due to the large amount of protein-level variation data reported by MAVEs, additional tools were needed to translate protein changes to codons. The protein changes were first mapped to the human GRCh38/hg38 genome assembly using the HGVS annotation strings provided in each mapped score set. Given this mapping, the changes were then translated to genomic coordinates using the HGVS interpretation tools available from the UCSC Genome Browser. Those coordinates were combined with the scores to form a “heatmap” track—a variant of the Browser Extensible Data (BED) filetype that stores the data for each heatmap as a single BED item. This yielded a single Genome Browser track that represents all single-substitution variants within each MaveDB score set. Filter settings on the track allow users to focus on specific score sets as desired.

### Integrating MaveDB data into the Ensembl Variant Effect Predictor

The Ensembl Variant Effect Predictor annotates variants described in genomic context, so for efficiency, MaveDB data was mapped to GRCh38 genome coordinates, aggregated into a single file and indexed with Tabix [80], using a custom NextFlow [81] pipeline. The pipeline downloaded assay results using the MaveDB API and combined them with mapping information. As Ensembl is an open resource, datasets submitted with licenses other than CC0 [82] were discarded. The Variant Recoder [83] was used to map variants described in protein space to genomic coordinates.

An Ensembl VEP plugin was developed to integrate MaveDB data into variant analyses [84]. This plugin searches the indexed MaveDB data file for matches to input variants, allowing filtering by the transcript specified in MaveDB and reporting scores, MaveDB identifiers, and relevant publications. The Ensembl VEP web interface provides direct links to MaveDB enabling easy access to all available information.

### Integrating MaveDB data into DECIPHER

The DECIPHER schema was extended and the aggregated MaveDB file created by Ensembl was loaded into the DECIPHER reference data database. Existing variant functional data displays were extended to show MaveDB data, including nucleotide and protein changes, scores and links to MaveDB for assay information and the publication for full details. An interactive decision tree was also developed to support the assessment of results [3].

### Integrating MaveDB data into the ClinGen Linked Data Hub

To integrate the MaveDB data into the ClinGen LDH, a MaveDBMapping document was created for each score set entry in the mapping files and added to the LDH as linked data for an LDH variant represented by the ClinGen Allele Registry canonical allele identifier. Because the ClinGen Allele Registry requires the use of standard human reference sequences (genome builds NCBI36, GRCh37, GRCh38 and transcripts from NCBI or Ensembl), each HGVS expression within the post-mapped objects from these score set entries was leveraged to either find the existing canonical allele identifier referenced in the score set entry or to register the variant with the ClinGen Allele Registry to obtain a new canonical allele identifier. MaveDBMapping documents were created by excerpting the MaveDB mapped scores object, score, MaveDB score set id (URN + entry number; e.g., urn:mavedb:00000001-a-1#1), captured provenance information (creation, modification, and publish dates), and a link back to the referenced MaveDB score set page.

## Supplementary Information

Additional file 1: Figures S1–6. This file contains additional figures that highlight the variant mapping workflow, score set metadata, and visualizations from the different genomics portals that have integrated mapped MAVE data. Fig. S1 MaveDB score set breakdown. Fig. S2 Variant mapping algorithm workflow. Fig. S3 Score set variant counts. Fig. S4 Downloading variant mappings via the MaveDB web interface. Fig. S5 Overview of MAVE data and visualization in the Genomics 2 Proteins Portal. Fig. S6 Overview of MaveDB mapping in LDH via the LDH-UI

Additional file 2: Mapping algorithm. This file contains a description of the MAVE sequence alignment and reference sequence selection steps of the mapping algorithm

Additional file 3: mave_g2p.csv. This file contains information about the number of mapped genes in the Genomics 2 Proteins Portal and their corresponding counts of single and double mutations

Additional file 4: Ensembl VEP supplementary information. This file contains a step-by-step guide for how to reproduce the output found in the Ensembl VEP figure

Additional file 5: Table S1. This file contains output from the score set metadata analysis. Table S1 Score set metadata statistics

Additional file 6: Running the mapping algorithm. The file contains a step-by-step guide for how to run the mapping algorithm

Additional file 7: Review history

## Data Availability

The datasets and code supporting the conclusions of this article are available in the dcd_mapping repository at https://github.com/ave-dcd/dcd_mapping [85]. The source code used in this study is stably archived using Zenodo at https://zenodo.org/records/14974961 [86]. The mapping files described in the manuscript can be downloaded at https://mavedb-mapping.s3.us-east-2.amazonaws.com/mappings_20250220.tar.gz.

## Declarations

### Review history

The review history is available as Additional file 7.

### Peer review information

Yang Li and Veronique van den Berghe were the primary editors of this article and managed its editorial process and peer review in collaboration with the rest of the editorial team.
