# Supplementary material for: Mapping MAVE data for use in human genomics applications
Source: Genome Biol. 2025 Jun 25;26:179. doi: 10.1186/s13059-025-03647-x (PMC12188674; doi:10.1186/s13059-025-03647-x)
Supplement: Supplementary file 1 — Additional file 1: Figures S1–6. This file contains additional figures that highlight the variant mapping workflow, score set metadata, and visualizations from the different genomics portals that have integrated mapped MAVE data. Fig. S1 MaveDB score set breakdown. Fig. S2 Variant mapping algorithm workflow. Fig. S3 Score set variant counts. Fig. S4 Downloading variant mappings via the MaveDB web interface. Fig. S5 Overview of MAVE data and visualization in the Genomics 2 Proteins Portal. Fig. S6 Overview of MaveDB mapping in LDH via the LDH-UI [file 13059_2025_3647_MOESM1_ESM.docx]

**
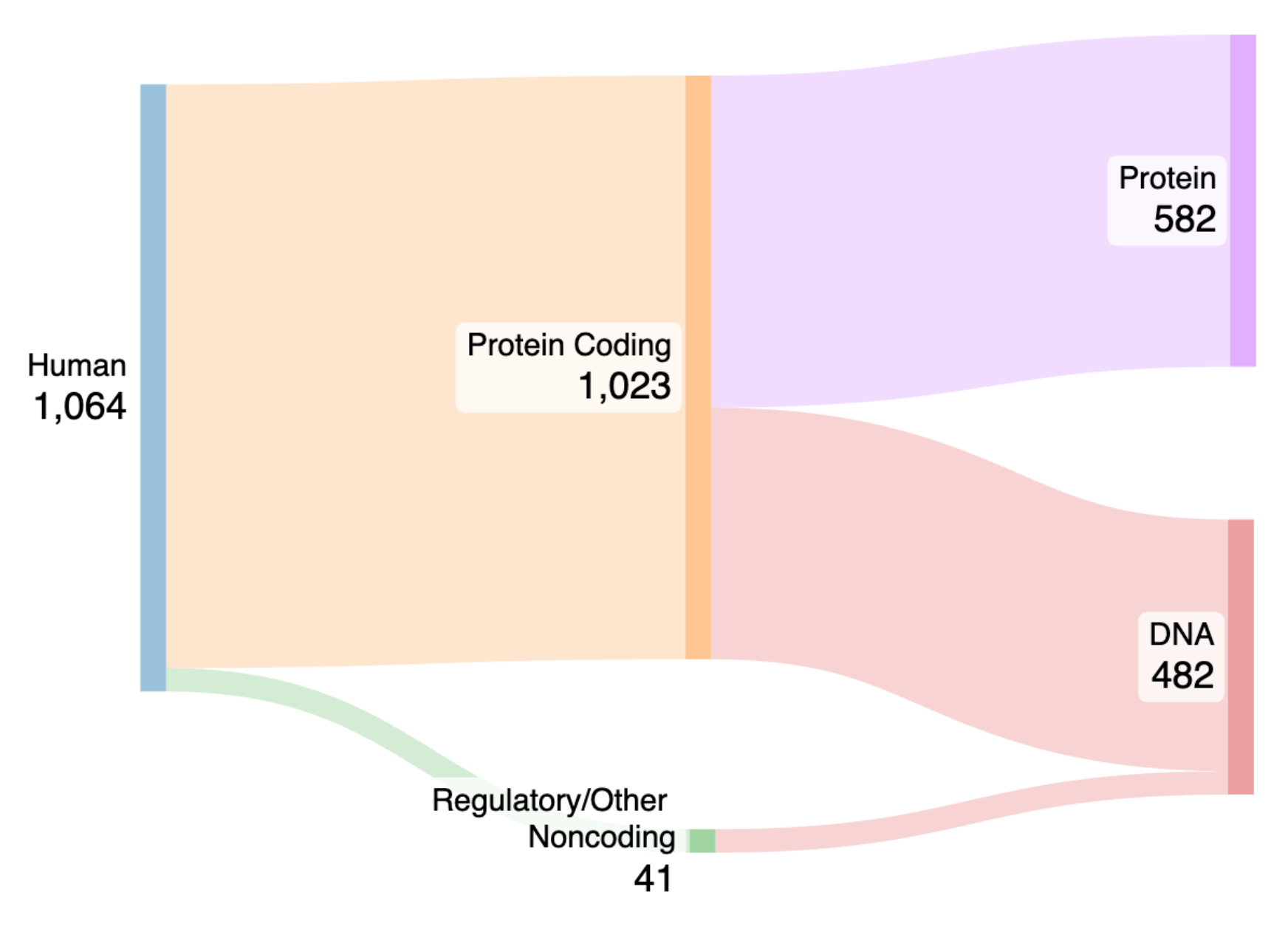
**

**Fig S1.** *MaveDB Score Set Breakdown*

A summary of the MAVE data from MaveDB that was used for validation of the mapping method. All score set entries in MaveDB are assigned an organism attribute (e.g. *Homo sapiens*, *Saccharomyces cerevisiae*). Score sets whose listed target organism was *Homo sapiens* (n = 1,064) were selected for testing of the mapping algorithm, and additional breakdowns describing the selected human score sets are presented. Made with SankeyMATIC.


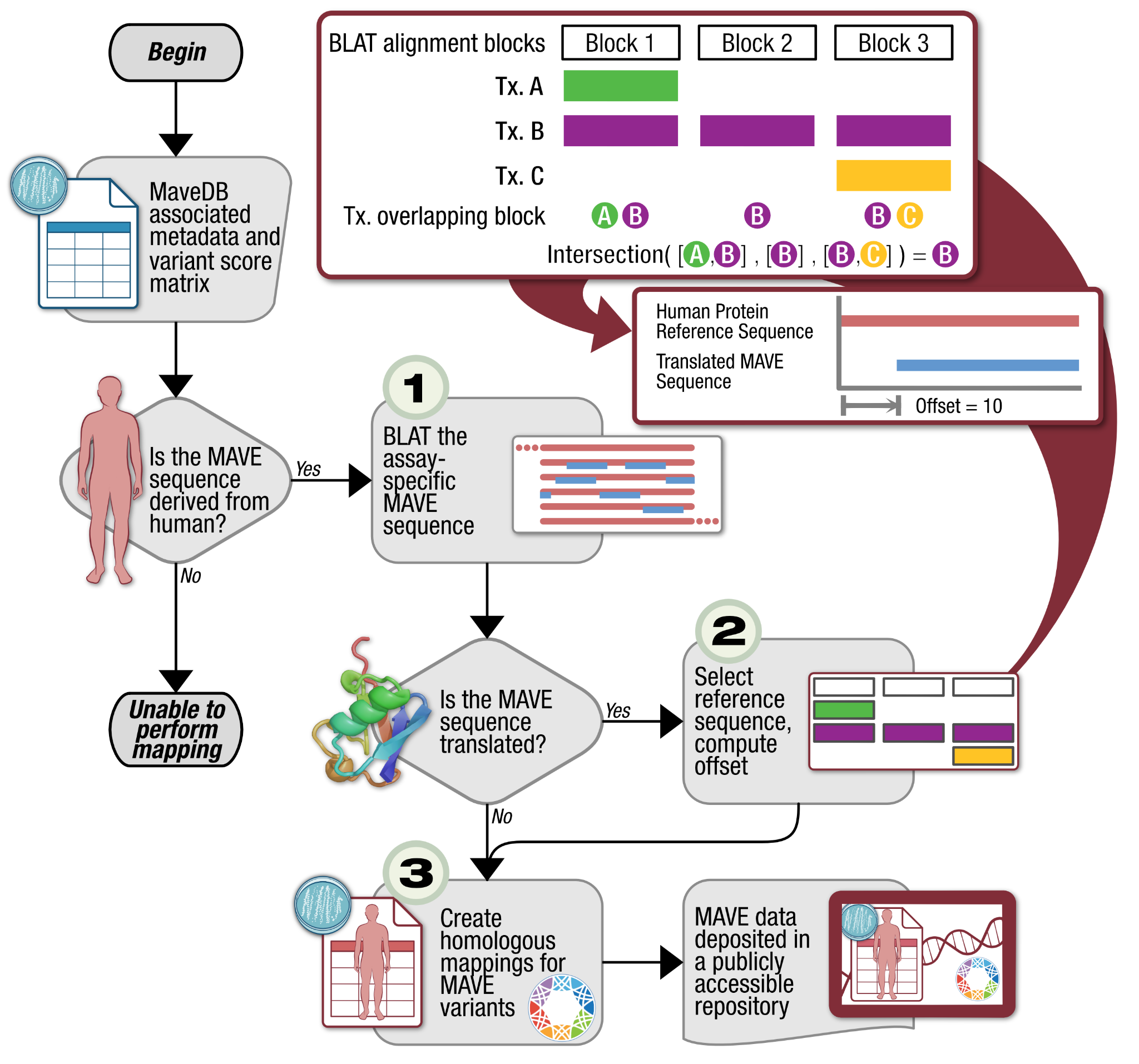


**Fig S2.** *Variant Mapping Algorithm Workflow*

A depiction of the MAVE variant mapping workflow. For a given entry in MaveDB whose listed target organism is *Homo sapiens*, the provided MAVE sequence is aligned to GRCh38 using BLAT, returning data including the chromosome number, gene symbol, and a set of genomic coordinates (**1**). If a score set describes a protein coding element, the outputted data can be supplied as a query to the Universal Transcript Archive (UTA) database, ultimately allowing for a RefSeq protein accession to be derived and for an offset to be computed (**2**). With a RefSeq sequence selected and offset calculated, the assayed variants in a MaveDB variant matrix are described with respect to their unique human reference sequence using the GA4GH Variation Representation Specification (VRS) (**3**). The resulting VRS objects are then annotated with descriptive metadata and integrated into specific score set JSON files. Lastly, the JSON files are gzipped and uploaded to a publicly-accessible s3 bucket to be available for downstream integration.

**
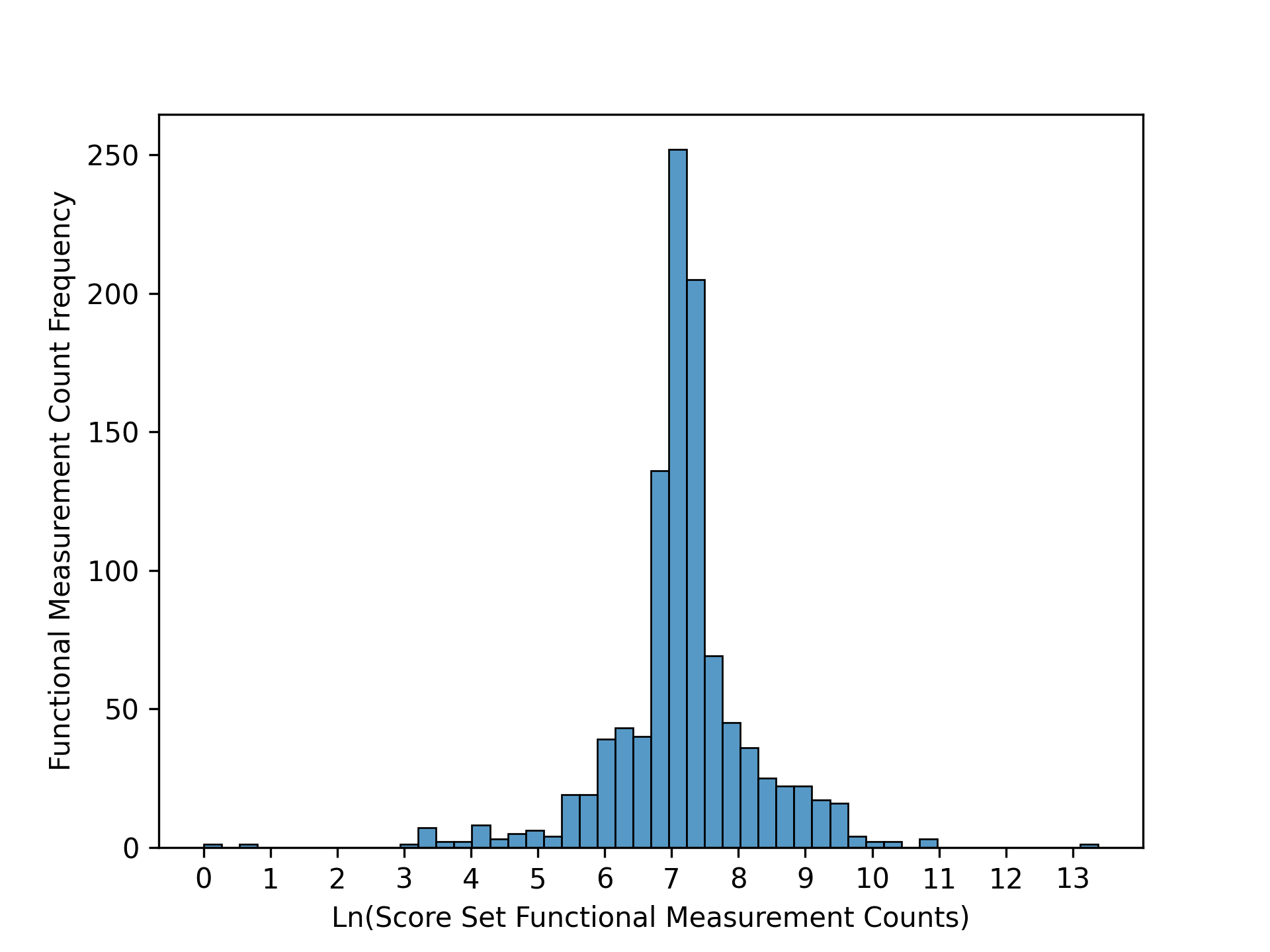
**

**Fig S3.** *Score Set Functional Measurement Counts*

The number of processed functional measurements per score set (ln-normalized) and the frequency of each functional measurement count.

**
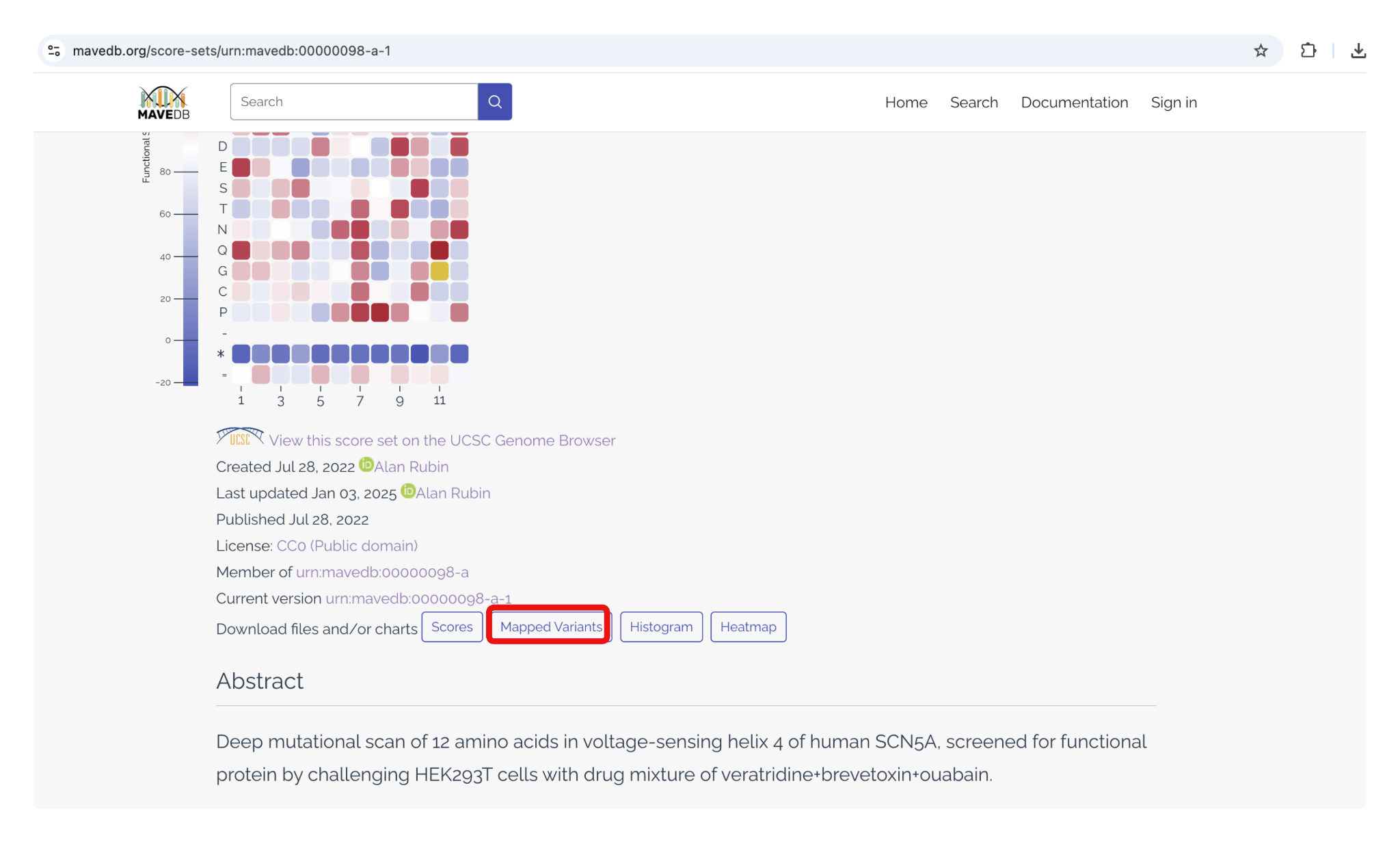
**

**Fig S4.** *Downloading Variant Mappings via the MaveDB Web Interface*

Mapped MaveDB score sets can be downloaded from the MaveDB web interface by clicking on the mapped variants box on the score set webpage (highlighted in the red box). Example displayed: score set urn:mavedb:00000098-a-1 (https://mavedb.org/score-sets/urn:mavedb:00000098-a-1).

**
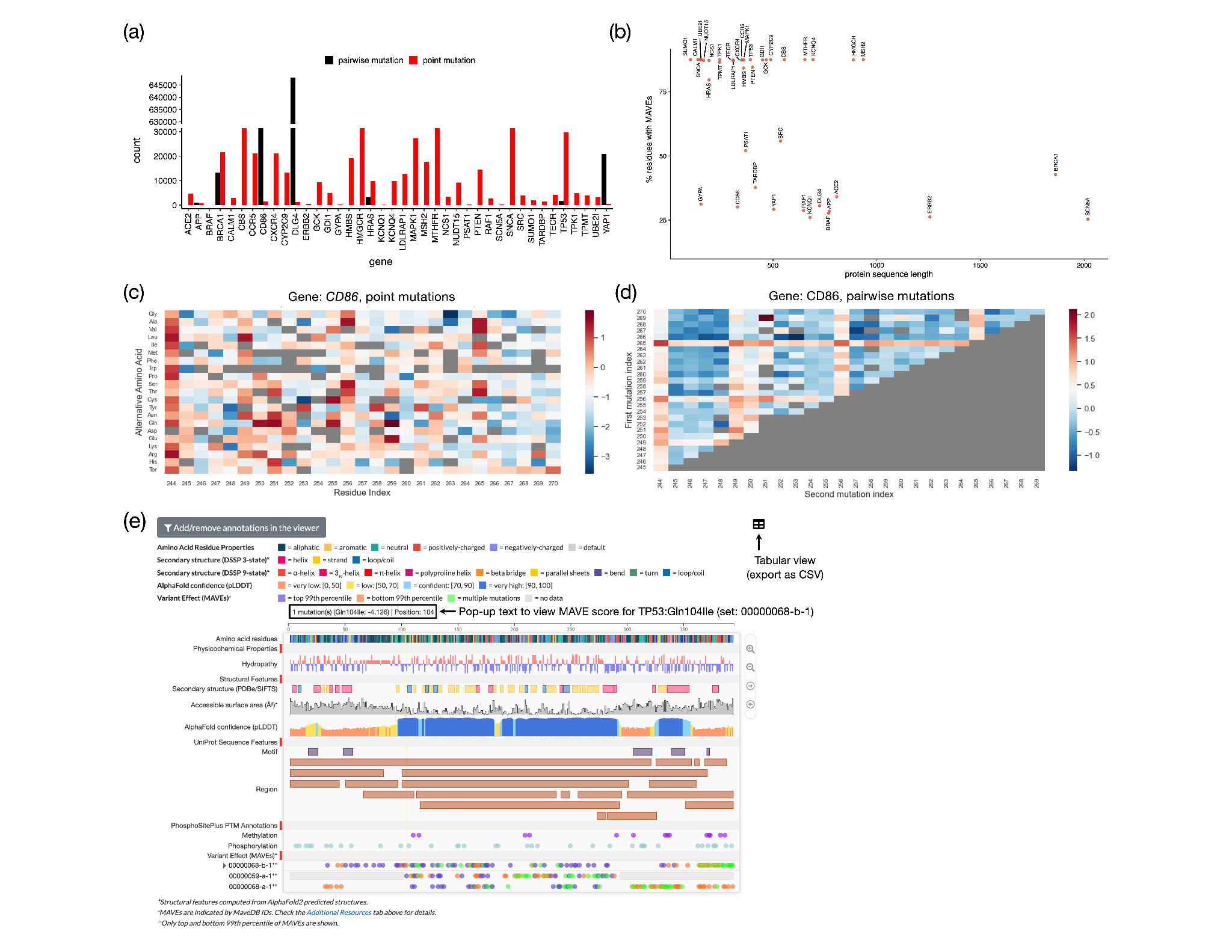
**

**Fig S5.** *Overview of MAVE Data and Visualization in the Genomics 2 Proteins Portal*

(**a**) Count of point mutations and pairwise mutations covered in MAVEs for 40 and 7 human genes out of 456 and 9 genes, respectively, for which MAVE data are available (full data available in Additional file 3). (**b**) Coverage of residues in MAVEs for each protein compared to the length of the sequence. (**c**) - (**d**) Heatmap visualization of MAVEs for gene and score set in the portal. (**e**) Annotation of protein sequence with mutations with top and bottom 99th percentile of MAVEs per score set in the context of protein physicochemical, structural and functional features.

**
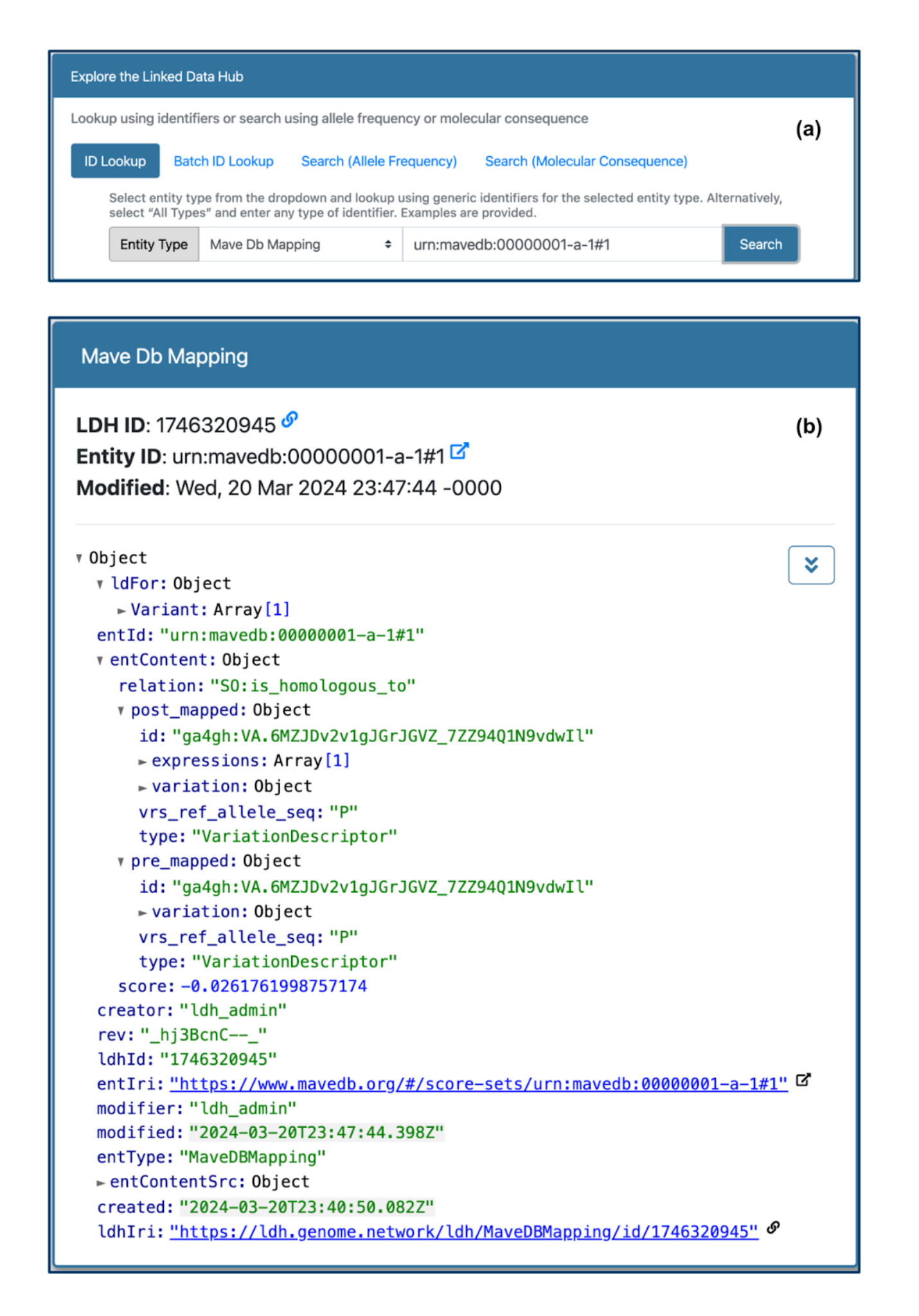
**

**Fig S6.** *Overview of MaveDB Mapping in LDH via the LDH-UI.*(**a**) ID lookup of MaveDB Mapping record by the LDH entity identifier - MaveDBMapping document’s entity ID (score set accession + “#” + variant number; e.g. urn:mavedb:00000001-a-1#1). (**b**) JSON formatted content including the linked variant, maveDB score, post and pre mapped data objects. Example displayed: https://ldh.clinicalgenome.org/ldh/ui/MaveDBMapping/id/urn%3Amavedb%3A00000001-a-1%231
