# Supplementary material for: Mapping MAVE data for use in human genomics applications
Source: Genome Biol. 2025 Jun 25;26:179. doi: 10.1186/s13059-025-03647-x (PMC12188674; doi:10.1186/s13059-025-03647-x)
Supplement: Supplementary file 2 — Additional file 2: Mapping algorithm. This file contains a description of the MAVE sequence alignment and reference sequence selection steps of the mapping algorithm [file 13059_2025_3647_MOESM2_ESM.docx]

# **Mapping Algorithm**

## *Alignment of Target Sequences to Human Genome*

Having extracted the necessary metadata from the MaveDB API, the initial step was to align target sequences to the human genome, allowing for the genomic coordinates of the examined sequences to be determined. To achieve this aim, all target sequences were run through BLAT against the GRCh38 human genome assembly. Depending on whether a target sequence was composed of nucleotides or amino acids, the BLAT query (q) argument was set to “dna” or “prot” to maximize the probability of returning high quality hits. In addition, the minimum score argument (minScore) was reduced to 20 from a default value of 30 to ensure that BLAT would return hits from short, specific target sequences.

After running BLAT, a series of steps were followed to ensure that suitable genomic coordinates were located. When running BLAT locally and outputting the results in a Pattern Space Layout (PSL) file, different “hits'' are reported at the chromosomal level (e.g. “chr3”). Within a hit are specific “HSP” (high-scoring pair) objects that describe regions of concordance between the queried sequence and the human genome. With the goal of ultimately selecting the correct HSP object, the correct chromosomal hit needs to be chosen first. To maximize this probability, when a UniProt accession was available for a score set, the UniProt accession was supplied as a compact uniform resource identifier (CURIE, e.g. uniprot:P12931) to the normalization method in the Variant Interpretation for Cancer Consortium (VICC) Gene Normalization Service, returning an HGNC consensus gene symbol and a chromosome number indicating where the gene occurs. Based on the chromosome number returned from the normalize method, the BLAT PSL file was filtered to only include the hit that contained the correct chromosome. If the filtered hit contained more than one HSP object, additional processing was required to select the correct genomic coordinates. First, the gene symbol corresponding to the provided UniProt accession was supplied to the search method within the Gene Normalization Service, returning location data, in genomic coordinates, from Ensembl and NCBI. Specifically, these two sources had “start” and “end” attributes describing the location where the supplied gene of interest occurs on a chromosome. Using the position indicated in the start attribute, the HSP object with the minimum distance to the start position was selected.

Having performed a series of filtering and validation steps to ensure that the genomic coordinates with the highest potential accuracy were selected, alignment data was stored in a custom data object. For each score set, the supplied data included: chromosome number, strand orientation, target name, target type, UniProt ID, percent coverage, percent identity, and a list reporting the genomic coordinates supplied by the selected HSP object.

While the alignment procedure performs well for protein coding score sets with UniProt accessions, its efficacy is potentially limited for regulatory/other noncoding score sets. Specifically, these score sets lack UniProt accessions and often have more descriptive target names without gene names (e.g. hYAP65 WW domain); the ability to extract consensus gene symbols for these score sets is limited, thereby resulting in a potential inability to perform the hit/HSP filtering procedure. In these instances, the top scoring hit reported by BLAT was selected, but the additional validation steps described previously were not performed.

## *RefSeq Transcript Selection and Offset Computation*

Having extracted genomic coordinates and other relevant information such as the chromosome number, there was sufficient data for selecting an appropriate human reference sequence for each score set. For the regulatory/other noncoding score sets that only reported genomic variation, the RefSeq chromosomal genomic reference sequence for GRCh38 was selected since the supplied genomic coordinates described locations on the sequence. However, for protein coding score sets that reported protein variants, a RefSeq protein reference sequence was needed, requiring use of the Biocommons UTA and SeqRepo databases and the associated Cool-Seq-Tool translation service.

First, using SeqRepo, a database that stores human reference sequences and links between different identifiers, chromosome numbers were converted to their GRCh38 RefSeq accession. Additionally, consensus gene symbols were derived by leveraging the Gene Normalization service that was utilized in the alignment procedure. Lastly, a query was run against UTA that extracted all the transcripts found within the genomic start and end coordinates, supplied by the HSP fragment, that were associated with the derived chromosome accession and gene symbol, and stored the identifiers in a list. This query was run for all coordinate pairs in the HSP list; for example, if an HSP object from a score set reported six fragments, six lists were generated. Once all lists were created and any non-coding transcript accessions were removed, the intersection of the lists was taken to produce a list of transcript accessions that were present across all fragments. If the method returned a nonempty list, the following prioritization ranking was followed for transcript selection: MANE Select, MANE Plus Clinical. If the list was empty, the lengths of all transcripts in the intersected list were taken, the longest transcript was chosen (with the first-published remaining transcript breaking ties), and the protein RefSeq accession associated with the selected transcript was found.

To determine the exact location of the provided target sequence within the RefSeq protein sequence, the target sequence, if DNA, was converted to protein using the standard codon table and the first 10 amino acids were extracted. Then, by accessing the RefSeq sequence using SeqRepo and using the find() method, the substring’s location was found; find() was also run on the entire converted protein sequence, producing a boolean that indicated if the *entire* target sequence was an exact substring of the RefSeq sequence. When the intersection procedure returned a non-RefSeq sequence, web scraping was performed to extract the canonical UniProt sequence and find() was again run twice. Lastly, the protein reference sequence, offset, score set accession, transcript and MANE status, and boolean were saved for downstream access.

There were a handful of score sets where discordance was observed between the provided target sequence and corresponding variant matrix. Assume a target sequence is a string of amino acid residues and reports Leucine at position 100. If the score matrix reports a substitution such as “p.Arg100His,” this would be an example of discordance as the expected reference amino acid would be Leucine instead of Arginine. In these cases, the variant matrix was parsed to assemble a dictionary reporting the expected reference amino acid at each position (ex. 1: M, 2: A, etc.). By comparing the expected amino acids from the dictionary to those at the corresponding positions in the target sequence, the correct start location in the target sequence was determined and the sequence offset was modified to reflect this position.

The general offset computed by the described procedure was applicable for protein variants in protein coding score sets. For genomic variants in protein coding score sets, the correct mapped position was found by determining the alignment block that the variant was found in and computing the distance between the variant position and the start (for positive strand) or end (for negative strand) genomic coordinate of the selected block, depending on the orientation of the target sequence. The same logic was followed for regulatory/other noncoding score sets with genomic variants that had only one alignment block.
