# Supplementary material for: Mapping MAVE data for use in human genomics applications
Source: Genome Biol. 2025 Jun 25;26:179. doi: 10.1186/s13059-025-03647-x (PMC12188674; doi:10.1186/s13059-025-03647-x)
Supplement: Supplementary file 4 — Additional file 4: Ensembl VEP supplementary information. This file contains a step-by-step guide for how to reproduce the output found in the Ensembl VEP figure [file 13059_2025_3647_MOESM4_ESM.docx]

## Annotating variants with MaveDB data using the Ensembl Variant Effect Predictor - an example

1. Navigate to the Ensembl VEP web interface: <https://www.ensembl.org/Tools/VEP>

2. Paste the following example variants into the ‘Input data’ entry field:

3 46373886 . G C

3 46373892 . G T

3 46373899 . G A

3 46373906 . C T

3 46373909 . G T

3. Scroll down to the ‘Functional Effect’ section and tick the square next to ‘MaveDB’.

4. Scroll down to the ‘Predictions’ section and tick the square next to ‘REVEL’.

5. Also in the ‘Predictions’ section, click the circle next to ‘CADD’ and select ‘CADD SNVs annotation file’.

6. Scroll to the bottom of the form and click the ‘Run >” button. This will send the analysis job to a queue.

7. When the job is marked as done (this should take under a minute), click on ‘View results’.

8. To filter by MaveDB score, use the ‘Filters’ tool to enter ‘MaveDB score > 1’ and click the ‘Add’ button. This score was selected for demonstration purposes; a score relevant to the assay should be chosen. Clicking on the MaveDB URN link will open the page on the MaveDB website with information on the assay which should be used to select an appropriate cutoff.

9. To optionally reduce the number of transcripts to be considered, use the ‘Filters’ tool to enter ‘MANE is defined’ and click the ‘Add’ button. (This reduces the results to show only one transcript for most genes, but some information can be lost.)
