# Supplementary material for: Mapping MAVE data for use in human genomics applications
Source: Genome Biol. 2025 Jun 25;26:179. doi: 10.1186/s13059-025-03647-x (PMC12188674; doi:10.1186/s13059-025-03647-x)
Supplement: Supplementary file 6 — Additional file 6: Running the mapping algorithm. The file contains a step-by-step guide for how to run the mapping algorithm [file 13059_2025_3647_MOESM6_ESM.docx]

# **Running the Mapping Algorithm**

## The steps below can be followed to run the mapping algorithm (see **Additional file 1: Fig. S2**) described in the manuscript.

## *Prerequisites*

- **Universal Transcript Archive (UTA)**: Please see the README (<https://github.com/biocommons/uta/blob/main/README.md>) for setup instructions. Users with access to Docker on their local devices can use the available Docker image; otherwise, start a relatively recent (version 14+) PostgreSQL instance and add data from the available database dump.
- **SeqRepo**: Please see the README (<https://github.com/biocommons/biocommons.seqrepo?tab=readme-ov-file#requirements>) for setup instructions. The SeqRepo data directory must be writeable; see specific instructions (https://github.com/biocommons/biocommons.seqrepo/blob/main/docs/store.rst) for more.
- **Gene Normalizer**: Please see the documentation (<https://gene-normalizer.readthedocs.io/0.3.0-dev1/install.html>) for data setup instructions.
- **blat**: Must be available on the local PATH and executable by the user. Otherwise, its location can be set manually with the BLAT_BIN_PATH env var. See the UCSC Genome Browser FAQ (<https://genome.ucsc.edu/FAQ/FAQblat.html#blat3>) for download instructions.
- **DCD_MAPPING_RESOURCES_DIR**: This parameter is used to specify the directory where the mapped files will be stored, along with the associated MaveDB score set and metadata files. The score set and metadata files, if not already present in this directory, will be accessed via the MaveDB API.

## *Installation*

Install from PyPi (<https://pypi.org/project/dcd-mapping/>).

Then, run the following command to install locally:

| python3 -m pip **install** dcd-**mapping** |
| --- |

## *Command Line Usage*

Use the dcd-map command with a scoreset URN, eg

| $ dcd-map “urn:mavedb:00000001-a-1” |
| --- |

Output is saved in the format <URN>_mapping_results_<ISO datetime>.json in the directory specified by the environment variable DCD_MAPPING_RESOURCES_DIR, or ~/.local/share/dcd-mapping by default.

Use dcd-map --help to see other available options.

## *Jupyter Notebook Usage*

The mapping algorithm can also be run in analysis notebooks:

| **from** os **import** environ **from** pathlib **import** Path  **from** dcd_mapping.main **import** map_scoreset_urn  # Set external resources. Configure based on location **of** available data. environ["GENE_NORM_DB_URL"] = "postgresql://postgres@localhost:5432/gene_normalizer" environ["UTA_DB_URL"] = "postgresql://uta_admin:uta@localhost:5432/uta/uta_20210129b" environ["BLAT_BIN_PATH"] = str(Path("blat").absolute()) environ["SEQREPO_ROOT_DIR"] = "/usr/local/share/seqrepo/2024-02-20"  environ["DCD_MAPPING_RESOURCES_DIR"] = str(Path("./mavedb_files").absolute())  # Map scoreset urn = "urn:mavedb:00000001-a-1" await map_scoreset_urn(urn) |
| --- |
