# Supplementary material for: Mapping MAVE data for use in human genomics applications
Source: Genome Biol. 2025 Jun 25;26:179. doi: 10.1186/s13059-025-03647-x (PMC12188674; doi:10.1186/s13059-025-03647-x)
Supplement: Supplementary file 7 — Additional file 7: Review history [file 13059_2025_3647_MOESM7_ESM.docx]

Review History

**First round of review**

**Reviewer 1**

**Were you able to assess all statistics in the manuscript, including the appropriateness of statistical tests used?**

Yes: The statistics shown in the manuscript are basic descriptive items to provide a general understanding about the scope of the tool presented. The manuscript presents a new tool to make the readouts of MAVE experiments collected through MaveDB standard and interoperable. In my view the study does not require a thorough statistical analysis and consequently some of the standard questions about statistics we reviewers are asked to answer in this form do not really apply in this case.

**Were you able to directly test the methods?**

No

**Comments to author:**

For the last decade, the meteoric development of genome sequencing technologies has reached enough cost-effectiveness so that it can be broadly implemented in research, but also in the clinic. However, its potential to improve clinical diagnosis and treatment remains very limited, the main obstacle being the difficulty to interpret the phenotypic consequences of the mutations found in patients.

Recently, multiplexed assays of variant effects (MAVE) have accomplished the remarkable feat of testing the functional effects of thousands of mutations at a time in artificial experimental systems, representing a formidable step to close the variant interpretation gap. These are generally very resource-intensive setups, difficult to scale and reproduce. To date, no more than 200 human genomic elements, including protein coding genes and regulatory elements have been studied with these means.

Beyond the potential caveats of the experimental systems not being faithful biological replicas of the human context of the disease, sometimes even resorting to non-human cellular contexts, thereby limiting the confidence at the mechanistic interpretation level, the readouts of these assays are invaluable for the basic understanding of genetic variation as well as for variant interpretation in the clinical setting, either directly or indirectly by means of benchmarking/validating other tools of more general applicability like in silico predictors or clinical variant interpretation systems.

The manuscript presents a method to standardize the readout of MAVE experiments collected in the MaveDB database, along with the results of applying the method to human score sets, comprising readouts linked to the assayed genetic variants. This is a very important necessary step to make these MAVE experimental outcomes widely accessible and interoperable for the community to make use of them.

The authors have already deployed their standardized variant annotations in a few widely used, publicly available genomics analysis portals, which renders the results of this effort particularly effective and immediate.

All in all, this reviewer considers that this is a commendable effort that can be of broad utility, particularly for those researchers working in the genomic angles of human disease, in particular those of us working more directly in understanding the role of germline as well as somatic variation (e.g. cancer, clonal hematopoiesis) in human health, aging and disease.

In general, the paper is well written and the steps of the methodology are carefully described. I would only like to point out a few minor comments:

- An important consideration about this type of effort is how easily it might be maintained and what are the potential caveats in rerunning as new data becomes available. I also wonder what the expected update frequency can be.

- Although this is a subjective take, I am not convinced that Fig 3 is the best attempt to convey the mapping algorithm workflow, it turns out to be quite cryptic and nonspecific at the same time. It really only made sense after reading the methods section in full.

- There are a few references to timelines and release dates that seem to require revision for the next publication, like e.g. in 218-220: "... we plan to update the Ensembl VEP REST and web interfaces to make open access MaveDB datasets available in our June 2023 release".


Typos and graphics:

- Fig 2A: Target Sequence Type
- Fig 2B: Dark color of green bar makes the number underneath difficult to read
- 304-305: "... and others will be provide …"

**Reviewer 2**

**Were you able to assess all statistics in the manuscript, including the appropriateness of statistical tests used?**

Yes: This paper reported percentages and was not comparing numbers to require any statistics.

**Were you able to directly test the methods?**

No

**Comments to author:**

This paper, titled Mapping MAVE data for use in human genomic applications, has nicely outlined the efforts of the AVE Alliance to standardize and disseminate functional data on variants of interest that could be related to human disease. It will be of broad interest to the field and a way to communicate the ways in which individuals can access this data for variant interpretation, but also for those in the genetics and broader scientific community who may be studying protein functional domains. Given that this paper is to introduce the broader community to databases that can be used to access the data, allowing linking between resources, I have a few comments and revisions listed below:

1 The results sections lays out the initial efforts for integration of MAVE data, including Genomics 2 Protein Portal, UCSC Genome Browser, ENSEMBL VEP, DECIPHER, ClinGen Linked Data hub, and Shariant. Positioning myself as a user that would like to access this data outlined, I found that many of the sites do not have direct user information on how to use, access, or understand the data. Many of the sites do not mention MAVE, AVE, or any other synonym in their sites data access or FAQs. This leaves a review to come across this by happenstance, rather than having direct instructions and guidance. I would recommend that all listed databases that have initially integrated MAVE DB include information that is searchable by a user, and that links that outline this from each source are included in the manuscript (whether the results or methods section).

To highlight this issue I have outlined what I found from a potential user perspective. G2P only has data if you happen to pick a gene with MAVE data (e.g. PTEN), there is no other way to know or search for genes with the Mave data from this site. Once you find a gene with data, there is a nice outline of the data with links back to MaveDB as the source. For the UCSC Genome Browser, the link in the paper is a specific instance from one of the authors, but going to the my projects, tracks section, I was not able to search and locate this track in the public hubs. I assume this track is intended for public user, so it would be good to add this information for the user before publication of the paper. For DECIPHER, the paper has a link to an example, but the DECIPHER sites Help and FAQ tabs do not have any information that is easily searchable or accessible to understand the incorporation of MaveDB, nor in their news section that typically indicates release of new materials with instructions. The ClinGen Linked Data Hub is also lacking any information on the inclusion of MaveDB data from its "Contents and Data" section as well as the API docs. Shariant cannot be accessed or viewed unless and individual creates a login. While this may still be a free resource, it is not as open access as others that don't require a login and it would be beneifical to either include and image, or indicate this requirement in the methods section.

2. There are several acronyms in the paper. Some have been defined while others, in particular in the methods section (e.g., VICC, CURIE) have not been defined. If this paper is intended for a broad audience, or even those that are new to these resources and work, it would be beneficial to either a) include a table of terminology that defines the acronyms, or b) define the acronym with the full name description at the first mention.

3. Figure 4, the figure legend does not indicate what is in the red boxes, please indicate.

4. Results section: The ClinGen Linked Data hub integration section indicates that information on MaveDB entries can be searched by the MAVE score set ID (e.g. urn:mavedb:00000001-a-1#1), when I went to the ClinGen LDH site (https://ldh.clinicalgenome.org/ldh/ui/) and searched by "all types" in the serach box this example I was returned "no matches." If the intent is for accessiblity to be only by API query and not on the front end of this website, please update to clarify this. Otherwise, the ClinGen LDH should update the search capacity to include the stated ID searches on the webste before publication of this manuscript.

**Reviewer 3**

**Were you able to assess all statistics in the manuscript, including the appropriateness of statistical tests used?**

Yes: no stat tests

**Were you able to directly test the methods?**

No

**Comments to author:**

This manuscript describes a method for mapping MaveDB variants to genome coordinates. MaveDB is or will become a database of crucial importance for medical diagnostics, so the topic is important and the data most likely useful.

However, mapping a protein variant through an alignment to the genome is in my opinion - and this is the first time that I'm writing this after having done reviews for 8 years - not what I'm used to see in Genome Biology, in terms of technical difficulty. To map a protein change to the genome from a protein sequence, one needs to align the protein sequence to the genome through an mRNA transcript, then walk along the alignment and count the nucleotides until one reaches the given position and output this position. MaveDB variants have the structure <proteinSequenceId>, <position>, <original amino acid>, <new amino acid>. While the manuscript uses various web services for some of these steps, none of these webservices are needed, a lookup in the RefSeq or UniProt tables would be sufficient. The manuscript uses UTA and R packages for some steps, but these are also not needed. To map from protein to genome, various tools already exist (none of them discussed in the manuscript), these could have been used or at least mentioned, the authors have not discussed at all the many, many existing software packages that map from protein to genome coordinates. The authors do mention the G2P database, which uses or collects data from databases who have used these tools. The authors present their own strategy without noting alternatives, e.g. HGVS is not needed, it's sufficient to frame the problem as "map protein sequence position X to genome given a protein/transcript and transcript/genome alignment" and while the manuscript solved this problem, I don't think a relatively straightforward and also common genomic coordinate conversion belongs into Genome Biology as an entire paper. It's not a method that people can use (and not a very efficient approach), it's not a new database and it's not a research paper either. Overall, the level of technical difficulty seems too low here, even if the resulting data is a useful for MaveDB users.

I looked at https://github.com/ave-dcd/dcd_mapping and this fits the picture. There are no instructions how anyone would be able to re-run this, where to start, what the steps are, what the output is. I searched and found this notebook https://github.com/ave-dcd/dcd_mapping/blob/main/notebooks/analysis/mavedb_mapping.ipynb. This looks like very low quality software to me. Basing results from clinical genetics labs on what looks like a throw-away notebook, without any comments or QC steps, with random function definitions thrown in, does not sound like a good idea to me. I had expected a normal piece of genomics software, at least a main short function, with a build step, fixed requirements, a command line program to run automated updates every few weeks, an update schedule for the future, not a Jupyter research log notebook full of pages and pages of uncommented code with random debugging output mixed into it.

**Authors Response**

**Point-by-point responses to the reviewers’ comments:**

**We thank the reviewers for their thoughtful and constructive feedback, which helped us improve the manuscript and the associated implementation and documentation. We think the resulting product is much more approachable as a result. Specific changes made in response to each of the reviewers’ comments are provided in-line below, with the reviewer comments in black and our responses in blue.**

Reviewer reports:

Reviewer #1: For the last decade, the meteoric development of genome sequencing technologies has reached enough cost-effectiveness so that it can be broadly implemented in research, but also in the clinic. However, its potential to improve clinical diagnosis and treatment remains very limited, the main obstacle being the difficulty to interpret the phenotypic consequences of the mutations found in patients.

Recently, multiplexed assays of variant effects (MAVE) have accomplished the remarkable feat of testing the functional effects of thousands of mutations at a time in artificial experimental systems, representing a formidable step to close the variant interpretation gap. These are generally very resource-intensive setups, difficult to scale and reproduce. To date, no more than 200 human genomic elements, including protein coding genes and regulatory elements have been studied with these means.

Beyond the potential caveats of the experimental systems not being faithful biological replicas of the human context of the disease, sometimes even resorting to non-human cellular contexts, thereby limiting the confidence at the mechanistic interpretation level, the readouts of these assays are invaluable for the basic understanding of genetic variation as well as for variant interpretation in the clinical setting, either directly or indirectly by means of benchmarking/validating other tools of more general applicability like in silico predictors or clinical variant interpretation systems.

The manuscript presents a method to standardize the readout of MAVE experiments collected in the MaveDB database, along with the results of applying the method to human score sets, comprising readouts linked to the assayed genetic variants. This is a very important necessary step to make these MAVE experimental outcomes widely accessible and interoperable for the community to make use of them.

The authors have already deployed their standardized variant annotations in a few widely used, publicly available genomics analysis portals, which renders the results of this effort particularly effective and immediate.

All in all, this reviewer considers that this is a commendable effort that can be of broad utility, particularly for those researchers working in the genomic angles of human disease, in particular those of us working more directly in understanding the role of germline as well as somatic variation (e.g. cancer, clonal hematopoiesis) in human health, aging and disease.

We thank the reviewer for comments recognizing the impact of this study and the research value of these data now that they are mapped onto common human references and available in publicly available genomics analysis portals.

In general, the paper is well written and the steps of the methodology are carefully described. I would only like to point out a few minor comments:

- An important consideration about this type of effort is how easily it might be maintained and what are the potential caveats in rerunning as new data becomes available. I also wonder what the expected update frequency can be.

We agree that it is important to ensure that our method can be applied to new data that is uploaded to MaveDB. Accordingly, we are working on developing a software package that will be used to apply our method to new score sets during the upload process. This will allow for a more persistent update frequency as mapped MAVE variants will be available along with the original submitted score set. We have discussed this new functionality in the revised version of our manuscript in the “Development of a Software Package for Mapping MaveDB Variants” section.

- Although this is a subjective take, I am not convinced that Fig 3 is the best attempt to convey the mapping algorithm workflow, it turns out to be quite cryptic and nonspecific at the same time. It really only made sense after reading the methods section in full.

We agree with your concerns about Figure 3 and have reworked the figure (now Figure 4 in the revised manuscript) to make it more accessible. Specific changes include more precise language describing our workflow and revised illustrations of each step.

- There are a few references to timelines and release dates that seem to require revision for the next publication, like e.g. in 218-220: "... we plan to update the Ensembl VEP REST and web interfaces to make open access MaveDB datasets available in our June 2023 release".

We have revised the manuscript to remove these references to integration plans, as initial integration of these data in each resource has been completed. Any remaining plans to extend this initial work are indicated only as future plans, without specific dates referenced.

Typos and graphics:

- Fig 2A: Target Sequence Type

- Fig 2B: Dark color of green bar makes the number underneath difficult to read

- 304-305: "... and others will be provide …"

We have corrected the two typos and updated the coloring for Figure 3B in the revised version of the manuscript.

Reviewer #2:

This paper, titled Mapping MAVE data for use in human genomic applications, has nicely outlined the efforts of the AVE Alliance to standardize and disseminate functional data on variants of interest that could be related to human disease. It will be of broad interest to the field and a way to communicate the ways in which individuals can access this data for variant interpretation, but also for those in the genetics and broader scientific community who may be studying protein functional domains. Given that this paper is to introduce the broader community to databases that can be used to access the data, allowing linking between resources, I have a few comments and revisions listed below:

1 The results sections lays out the initial efforts for integration of MAVE data, including Genomics 2 Protein Portal, UCSC Genome Browser, ENSEMBL VEP, DECIPHER, ClinGen Linked Data hub, and Shariant. Positioning myself as a user that would like to access this data outlined, I found that many of the sites do not have direct user information on how to use, access, or understand the data. Many of the sites do not mention MAVE, AVE, or any other synonym in their sites data access or FAQs. This leaves a review to come across this by happenstance, rather than having direct instructions and guidance. I would recommend that all listed databases that have initially integrated MAVE DB include information that is searchable by a user, and that links that outline this from each source are included in the manuscript (whether the results or methods section).

To highlight this issue I have outlined what I found from a potential user perspective. G2P only has data if you happen to pick a gene with MAVE data (e.g. PTEN), there is no other way to know or search for genes with the Mave data from this site. Once you find a gene with data, there is a nice outline of the data with links back to MaveDB as the source. For the UCSC Genome Browser, the link in the paper is a specific instance from one of the authors, but going to the my projects, tracks section, I was not able to search and locate this track in the public hubs. I assume this track is intended for public user, so it would be good to add this information for the user before publication of the paper. For DECIPHER, the paper has a link to an example, but the DECIPHER sites Help and FAQ tabs do not have any information that is easily searchable or accessible to understand the incorporation of MaveDB, nor in their news section that typically indicates release of new materials

with instructions. The ClinGen Linked Data Hub is also lacking any information on the inclusion of MaveDB data from its "Contents and Data" section as well as the API docs. Shariant cannot be accessed or viewed unless and individual creates a login. While this may still be a free resource, it is not as open access as others that don't require a login and it would be beneifical to either include and image, or indicate this requirement in the methods section.

We agree that it is important for the listed databases to include documentation on the integrated MaveDB data and include links to this information in the manuscript. Accordingly, we have made the following additions, as noted below:

- The list of genes in G2P with MAVE data can be viewed under the “Protein Features” tab on the statistics page at <https://g2p.broadinstitute.org/stats>. G2P has also been recently updated to include the average functional effect scores at different positions in the MAVE sequence (e.g. the average score at position 218 for CYP2C9 is 0.923), as seen in the red boxes.

- We have added a new session to the UCSC Genome Browser at <https://genome.ucsc.edu/s/mcline/MaveDB>, as described in the manuscript. For accessibility, we have added this session to the UCSC Genome Browser’s Public Session gallery at <https://genome.ucsc.edu/cgi-bin/hgPublicSessions>. Each mapped score set in MaveDB also includes a link to the associated mappings track on the UCSC Genome Browser (see example below for score set 68-b-1), as shown in the red box.

- Information and instructions on how to access MAVE data via DECIPHER has been added to the DECIPHER homepage under “Latest news” at <https://www.deciphergenomics.org/> and news page <https://www.deciphergenomics.org/about/news> . This information is shown below and is indicated by the red boxes.

- Ensembl VEP contains instructions on how to incorporate MAVE data through the command line tool at <https://useast.ensembl.org/info/docs/tools/vep/script/vep_plugins.html#mavedb>. There are additional instructions on the web interface at <https://useast.ensembl.org/Homo_sapiens/Tools/VEP?db=core> and the REST API at <https://rest.ensembl.org/documentation/info/vep_region_get>.

- The ClinGen Linked Data Hub has been updated so the number of “Mave Db Mapping” objects is visible under the LDH stats table. MAVE variants can be queried through LDH under the “Explore the Linked Data Hub” section.

- The Shariant platform can only be accessed by Australian and New Zealand laboratories conducting clinical grade-testing. We have indicated this access restriction at the end of the Shariant section.

2. There are several acronyms in the paper. Some have been defined while others, in particular in the methods section (e.g., VICC, CURIE) have not been defined. If this paper is intended for a broad audience, or even those that are new to these resources and work, it would be beneficial to either a) include a table of terminology that defines the acronyms, or b) define the acronym with the full name description at the first mention.

We have added an *abbreviations* section after the methods section that describes the meaning of each acronym.

3. Figure 4, the figure legend does not indicate what is in the red boxes, please indicate.

We have added text to the figure description in the revised manuscript indicating that the content in the red boxes presents data from MaveDB in G2P and Ensembl VEP.

4. Results section: The ClinGen Linked Data hub integration section indicates that information on MaveDB entries can be searched by the MAVE score set ID (e.g. urn:mavedb:00000001-a-1#1), when I went to the ClinGen LDH site (https://urldefense.com/v3/__https://ldh.clinicalgenome.org/ldh/ui/__;!!KGKeukY!3AVfEhVrBORhDYW1yRHEJNNmKyCSJgaxlf2AKzqCvOA5yF5RNYN4O12Fa0vgNDs4NztUHugIn1dGkyBoDS8$ ) and searched by "all types" in the serach box this example I was returned "no matches." If the intent is for accessiblity to be only by API query and not on the front end of this website, please update to clarify this. Otherwise, the ClinGen LDH should update the search capacity to include the stated ID searches on the webste before publication of this manuscript.

We appreciate this comment and agree with the need to make data from the ClinGen LDH integration more accessible. The ClinGen LDH website (https://ldh.clinicalgenome.org/ldh/ui/) has been updated so that users can query “Mave Db Mapping” objects under the “Explore the Linked Data Hub” section, as illustrated above. Queries can be run in this section using the appropriate MaveDB ID (e.g. urn:mavedb:00000001-a-1#1) or LDH ID (e.g. 1746320945). The ClinGen Linked Data Hub integration section of the manuscript has been updated to illustrate this feature. Importantly, we believe this addition will make MAVE data more accessible for downstream users via the ClinGen Linked Data Hub.

Reviewer #3: This manuscript describes a method for mapping MaveDB variants to genome coordinates. MaveDB is or will become a database of crucial importance for medical diagnostics, so the topic is important and the data most likely useful.

We agree with the reviewer about the importance of the MaveDB resource, and think that this study fills an important gap to making those data accessible for genomics research applications.

This effort was a product of our coordinated effort between numerous experts in MAVE experiment data, genomic databases (including the maintainers of MaveDB), and variant representation under the auspices of the Data Coordination and Dissemination work stream of the Atlas for Variant Effects (AVE) Alliance. As the corresponding authors on this effort, we are excited about the work presented in this manuscript, which helped us overcome a key challenge in the reusability and interoperability of MAVE data due to the complex nature of these experiments and their associated data.

However, mapping a protein variant through an alignment to the genome is in my opinion - and this is the first time that I'm writing this after having done reviews for 8 years - not what I'm used to see in Genome Biology, in terms of technical difficulty. To map a protein change to the genome from a protein sequence, one needs to align the protein sequence to the genome through an mRNA transcript, then walk along the alignment and count the nucleotides until one reaches the given position and output this position.

While we appreciate the constructive intentions behind the reviewer’s emphatic criticism, we must point out that–unlike the other reviewers–this reviewer has mischaracterized the nature of the work as a simple exercise in mapping from protein changes to genomic changes. As discussed at length in the paper introduction, the focus of this work is not protein to genome mapping, but mapping from assay-specific target sequences (specified at the DNA or protein level) to human reference sequences. An excerpt from the manuscript introduction succinctly describes the challenge:

*These variants are represented with respect to a* target sequence *uploaded by the submitter; however, as this target sequence is not described with respect to human reference sequence sets (e.g. Ensembl/GENCODE*[*(17)*](https://paperpile.com/c/cmSpLn/wFXe)*, RefSeq[(17,18)](https://paperpile.com/c/cmSpLn/wFXe+rjIU), GRC genome assemblies), a challenge emerges concerning the standardized representation of variation.*

We also discuss supplied target sequences in Figure 1, which describes the primary goal of our work and reads:

*MAVE variants are described with respect to supplied target sequences, but the absence of linkages to human reference sequences limits the interoperability of MAVE data with human genomics applications (left). To overcome this limitation, we have developed a method to map MAVE variants to their corresponding human reference sequences (middle).*

We have added text to this caption clarifying that these sequences are *custom, user-submitted* target sequences, and have additionally created an illustrated overview of the challenges faced in the mapping process as a new Figure 2 to better orient readers to the challenges addressed in this work.

MaveDB variants have the structure <proteinSequenceId>, <position>, <original amino acid>, <new amino acid>.

This is factually incorrect. The relevant excerpts about the structure of MaveDB variants are described in the paper introduction:

*For a given entry in MaveDB, a MAVE dataset describes sequence changes in MAVE-HGVS*[*(15)*](https://paperpile.com/c/cmSpLn/ByAq)*, a format similar to the Human Genome Variation Society (HGVS) variant nomenclature*[*(16)*](https://paperpile.com/c/cmSpLn/qbrOL)*. MAVE-HGVS variants are then associated with their respective functional scores.*

*Specifically, while variants in HGVS are described in relation to accepted reference sequences (e.g. NP_003997.1:p.Trp24Cys), the vast majority of variants in MAVE score sets in MaveDB are described only in the context of the assayed sequence.*

While the manuscript uses various web services for some of these steps, none of these webservices are needed, a lookup in the RefSeq or UniProt tables would be sufficient. The manuscript uses UTA and R packages for some steps, but these are also not needed. To map from protein to genome, various tools already exist (none of them discussed in the manuscript),

these could have been used or at least mentioned, the authors have not discussed at all the many, many existing software packages that map from protein to genome coordinates.

As discussed above, we have added Figure 2 to the manuscript to illustrate the challenges faced in the mapping of MAVE experimental data to human reference sequences, and expect this will help clarify to the reviewer and Genome Biology readership the challenges we needed to address in this work. We agree with the value of not reinventing the wheel and contributing to community tools when appropriate. We assure the reviewer that we reuse existing tools where relevant to this activity, including the UCSC Blat utility, Biocommons tools (including UTA), and GA4GH VRS-Python. In places where we felt it prudent to create additional convenience methods, we have deployed web services to aid in reproducibility; though these, too, are also locally installable and can be used via Python APIs in lieu of RESTful HTTPS. We do not know what R packages the reviewer is referring to regarding our mapping process; the only R packages used in this work were dplyr, ggplot2, and magrittr, to generate a figure illustration for the revised figure 3B.

The authors do mention the G2P database, which uses or collects data from databases who have used these tools. The authors present their own strategy without noting alternatives, e.g. HGVS is not needed, it's sufficient to frame the problem as "map protein sequence position X to genome given a protein/transcript and transcript/genome alignment" and while the manuscript solved this problem, I don't think a relatively straightforward and also common genomic coordinate conversion belongs into Genome Biology as an entire paper.

As discussed above, the variants in the MaveDB resource are specified using MAVE-HGVS, so we must respectfully disagree with the reviewer that HGVS translation software (using pre-existing HGVS translation software, from Biocommons) is not needed. Also, we must again respectfully point out to this reviewer that our work was a necessary advancement for making these data accessible to the community, as evidenced by the many integrations discussed in the manuscript.

It's not a method that people can use (and not a very efficient approach), it's not a new database and it's not a research paper either. Overall, the level of technical difficulty seems too low here, even if the resulting data is a useful for MaveDB users.

We appreciate this reviewer’s concerns about reusability of our work, and have made several revisions to documentation and packaging of our code, discussed below. We also want to underscore that the key outcomes from our study are the 2.5 million mapped variants now accessible on human reference sequences, and the integration of these mappings into community genomic research platforms, including the UCSC Genome Browser, DECIPHER, Ensembl VEP, the ClinGen Linked Data Hub, and the Broad Genomics 2 Proteins Portal.

I looked at [https://urldefense.com/v3/__https://github.com/ave-dcd/dcd_mapping__;!!KGKeukY!3AVfEhVrBORhDYW1yRHEJNNmKyCSJgaxlf2AKzqCvOA5yF5RNYN4O12Fa0vgNDs4NztUHugIn1dGQEtCvJ8$](https://urldefense.com/v3/__https:/github.com/ave-dcd/dcd_mapping__;!!KGKeukY!3AVfEhVrBORhDYW1yRHEJNNmKyCSJgaxlf2AKzqCvOA5yF5RNYN4O12Fa0vgNDs4NztUHugIn1dGQEtCvJ8$) and this fits the picture. There are no instructions how anyone would be able to re-run this, where to start, what the steps are, what the output is. I searched and found this notebook [https://urldefense.com/v3/__https://github.com/ave-dcd/dcd_mapping/blob/main/notebooks/analysis/mavedb_mapping.ipynb__;!!KGKeukY!3AVfEhVrBORhDYW1yRHEJNNmKyCSJgaxlf2AKzqCvOA5yF5RNYN4O12Fa0vgNDs4NztUHugIn1dG7FZqasw$](https://urldefense.com/v3/__https:/github.com/ave-dcd/dcd_mapping/blob/main/notebooks/analysis/mavedb_mapping.ipynb__;!!KGKeukY!3AVfEhVrBORhDYW1yRHEJNNmKyCSJgaxlf2AKzqCvOA5yF5RNYN4O12Fa0vgNDs4NztUHugIn1dG7FZqasw$) . This looks like very low quality software to me. Basing results from clinical genetics labs on what looks like a throw-away notebook, without any comments or QC steps, with random function definitions thrown in, does not sound like a good idea to me.

We believe that the reviewer language used here is unnecessarily harsh, though we appreciate the reviewer’s intent: to encourage development of approachable analysis documentation for reproducibility. We have spent time clarifying the content of these notebooks to improve readability for people to better understand the methods employed to map variants from the MaveDB resource to human reference sequences.

Instructions on how anyone may reproduce our analysis may be found here: <https://go.osu.edu/mavedb-mapping-analysis-readme>. As indicated in those instructions, the best place to start is this notebook: <https://go.osu.edu/mavedb-mapping>. The steps in the notebook now directly align with the illustrations and descriptive text in revised Figure 4 of the manuscript. In addition to the description of these output files as provided in the manuscript, we have generated a JSON Schema (<https://go.osu.edu/mavedb-mapping-json-schema>) that can be used to computationally validate and reason on the structure of these files.

I had expected a normal piece of genomics software, at least a main short function, with a build step, fixed requirements, a command line program to run automated updates every few weeks, an update schedule for the future, not a Jupyter research log notebook full of pages and pages of uncommented code with random debugging output mixed into it.

We agree with the reviewer that a packaged software product would improve the reproducibility and generalizability of our work, and expect this software may be useful in other contexts. Therefore, in addition to our improvements in the readability and usability of our analysis notebooks described above, we have also developed a command-line tool that allows users to run our pipeline on individual score sets, and we are in the process of deploying this for use on the MaveDB server for new submissions. The software code is open-source and documented at <https://go.osu.edu/mavedb-mapping-readme>. The software is readily available for installation from the Python Package Index (PyPI) using standard python package installation tools (i.e. “pip install”), as described in the software documentation.

**Second round of review**

**Reviewer 1**

The method and tools presented in the manuscript add much value to the MaveDB repository. I am looking forwards to seeing whether this public resource takes off and gets more widely employed by the community, potentially leading to a rich resource for genomic studies. The work presented in the manuscript is a step in the right direction.

As far as this reviewer is concerned there are no further comments: the authors have satisfactorily responded to the review.

**Reviewer 3**

This is important data, as I wrote. I hope the authors will indeed run this convoluted pipeline again. Our disagreement is about how this was implemented.

> As discussed at length in the paper introduction, the focus of this work is not

> protein to genome mapping, but mapping from assay-specific target sequences (specified

> at the DNA or protein level) to human reference sequences.

"protein to genome mapping" does not mean that the protein is a 100% match. I think I made this clear.

I repeat what I wrote in the my critique: to map a position from a query sequence to the genome, one just walks along the two pairwise alignments (see below). Yes, the query sequence can be different, a "assay-specific target sequence" how you call it. I did not write that the protein query was identical to the genome. It is obvious that the target sequence is not identical to the genome. I never wrote that that the protein is the exact 100% translation of the genome. It can be any protein (see below).

> We have added text to this caption clarifying that these sequences are custom, usersubmitted target sequences,

Yes, I was aware of this. Because it's the same everywhere in genomics. Apparently you are not aware of this, but RefSeq sequences are custom sequences. PDB sequences don't match the genome. UniProt sequences do not match the genome, yet UniProt, PDB and RefSeq and any good HGVS resolver maps to the genome from these proteins through alignments (Bioconductor/R is an exception). CRAVAT maps between PDB and genome. LS-SNP maps from PDB proteins to dbSNP, so the genome. There must be dozens of other tools where the mapping from protein to genome is handled in two paragraphs. 3% of RefSeqs have indels, sometimes long ones. The basic underlying technical problem is exactly the same as with your "assay-specific target sequences": the sequences don't match, so you could have repeated what Mutalyzer or the hgvs packages do, just using your custom protein sequences and special protein-Refseq alignments (see below).

>> MaveDB variants have the structure <proteinSequenceId>, <position>, <original amino

>> acid>, <new amino acid>.

> This is factually incorrect. The relevant excerpts about the structure of MaveDB variants

> are described in the paper introduction:

> For a given entry in MaveDB, a MAVE dataset describes sequence changes in MAVEHGVS(15), a format similar to the Human Genome Variation

> Society (HGVS) variant

> Specifically, while variants in HGVS are described in relation to accepted reference

> sequences (e.g. NP_003997.1:p.Trp24Cys), the vast majority of variants in MAVE score

> sets in MaveDB are described only in the context of the assayed sequence.

First you write that what I wrote is factually incorrect, then you write basically the same thing: that your changes are defined on a protein sequence. That's what I wrote. You seem that think that all protein sequences in databases match the genome. This is the misunderstanding here. But they don't, not always, and a lot of software already exists to solve this problem, map protein sequences that differ from the genome to the genome.

> Also, we must again respectfully point out to this reviewer that our work was a necessary

> advancement for making these data accessible to the community, as evidenced by the

> many integrations discussed in the manuscript.

I totally agree that this work is important, I wrote that myself in the very first sentence of my critique.

What I criticize is the technical difficulty and the way this is implemented. You could have used just mapped from your protein to the Refseq sequence through an alignment, then use mutalyzer's position converter to go to the genome and be done with it. After having checked, I agree, not with R, you could have not used "proteinToGenome()" in Bioconductor, as that is not using an alignment (my fault). But in your paper, there is a lot of text, a special python package, various APIs, a UTA, and more dependencies. I have never heard of Gene Normalizer before, it looks like it could be a two column text file. All this overengineering will make the pipeline very difficult to run in the future or for someone else. Reading this paper, a student a bioinformatics could get the idea that the task target-protein-sequence + position -> genome is a hard problem.

All data that is needed is the RefSeq alignment to the genome from NCBI as a GFF3 and a BLAST or BLAT alignment of your MaveDB protein or nucleotide sequences to RefSeq transcript annotated in MaveDB. All source code that is needed is a single Python loop to do the nucleotide or amino acid counting along these two alignments. I'm not sure how such a simple task is taking up pages of methods and dozens of acronyms.

I agree that one problem if you want to use mutalyzer instead or transvar or hgvs, you still need to map from protein to RefSeq through an alignment, but that's not rocket science.

**Authors Response**

**Point-by-point responses to the reviewers’ comments:**

**We thank the reviewers again for their thoughtful and constructive feedback. Specific changes made in response to each of the reviewers’ comments are provided in-line below, with the reviewer comments in black and our responses in blue.**

**Reviewer #1**

The method and tools presented in the manuscript add much value to the MaveDB repository. I am looking forwards to seeing whether this public resource takes off and gets more widely employed by the community, potentially leading to a rich resource for genomic studies. The work presented in the manuscript is a step in the right direction.

As far as this reviewer is concerned there are no further comments: the authors have satisfactorily responded to the review.

We thank the reviewer for their positive comments and appreciate the sentiment about the value this work adds to the MaveDB repository. We are excited to realize, through this study, the integration of these data in several downstream clinical and research genomics resources, and agree this is an important step in the direction of routinely applying these data to downstream genomic studies.

**Reviewer #3**

This is important data, as I wrote. I hope the authors will indeed run this convoluted pipeline again. Our disagreement is about how this was implemented.

We thank the reviewer for acknowledging the importance of the data we have generated. We realize that our work left the reviewer with an impression of an unnecessarily complex implementation, and we have taken this suggestion to heart in revising the structure of our manuscript to improve the clarity of our work. However (as the reviewer acknowledges below during the course of this secondary review) the challenges presented by the MAVE data prevent us from using a single, off-the-shelf solution, underscoring the value of our developed process.

> As discussed at length in the paper introduction, the focus of this work is not

> protein to genome mapping, but mapping from assay-specific target sequences (specified

> at the DNA or protein level) to human reference sequences.

"protein to genome mapping" does not mean that the protein is a 100% match. I think I made this clear.

I repeat what I wrote in the my critique: to map a position from a query sequence to the genome, one just walks along the two pairwise alignments (see below). Yes, the query sequence can be different, a "assay-specific target sequence" how you call it. I did not write that the protein query was identical to the genome. It is obvious that the target sequence is not identical to the genome. I never wrote that that the protein is the exact 100% translation of the genome. It can be any protein (see below).

We appreciate the clarification from the reviewer that they recognize that not all submitted MAVE sequences match 100% to the human genome. We agree with the reviewer that walking across pairwise alignments is a valid strategy for coordinate mapping, and indeed this is a key component of our work, leveraging the established community BLAT tool to generate the alignments for this step. We remind the reviewer that our work additionally needed to handle the challenges of selection of a representative transcript (and corresponding protein sequence) from such alignments, re-normalization and validation of variants described on both reference and non-reference sequences, and representation of variant provenance to enable contextual reuse of MAVE data in downstream resources. These points have been overlooked in the reviewer comments but constitute a substantial portion of our methods and the value of the resulting dataset.

> We have added text to this caption clarifying that these sequences are custom, usersubmitted target sequences,

Yes, I was aware of this. Because it's the same everywhere in genomics. Apparently you are not aware of this, but RefSeq sequences are custom sequences. PDB sequences don't match the genome. UniProt sequences do not match the genome, yet UniProt, PDB and RefSeq and any good HGVS resolver maps to the genome from these proteins through alignments (Bioconductor/R is an exception). CRAVAT maps between PDB and genome. LS-SNP maps from PDB proteins to dbSNP, so the genome. There must be dozens of other tools where the mapping from protein to genome is handled in two paragraphs. 3% of RefSeqs have indels, sometimes long ones. The basic underlying technical problem is exactly the same as with your "assay-specific target sequences": the sequences don't match, so you could have repeated what Mutalyzer or the hgvs packages do, just using your custom protein sequences and special protein-Refseq alignments (see below).

We took care to describe these as “custom, user-submitted” sequences to specifically convey the differences between these and *community reference* sequences, such as those in RefSeq and UniProt. While this paragraph opens by suggesting there is no practical difference between these datasets, we recognize that the reviewer understands the additional challenges posed by user-submitted sequences (which lack the centrally accessible, pre-computed alignment data provided by community references). Specifically, we appreciate the recommendation here to use “custom protein sequences and special protein-Refseq alignments” with existing software platforms such as the hgvs package.

As maintainers of the Biocommons hgvs package, we agree with the notion of reusing these technologies where appropriate. As described in the manuscript methods, the Biocommons seqrepo package (used by hgvs) was key software in this study for registering and retrieving the submitted custom sequences, addressing this exact suggestion from the reviewer. The hgvs package itself was subsequently used to generate HGVS nomenclature descriptions for the post-mapped variants for integration in downstream tools. “Special protein-RefSeq alignments” do not account for sequences that do not align to RefSeq accessions, such as MAVEs targeting regulatory elements, as discussed in the manuscript. Our strategy generalizes this solution proposed by the reviewer, by aligning to the genome first and (when appropriate) selecting compatible RefSeq transcripts from the alignment data. Both strategies require target sequence alignment and (when appropriate) transcript selection strategies, and we considered the generalizable approach best suited to working across the entire collection of MaveDB scoresets.

We acknowledge the reviewer’s point that we have provided exhaustive detail in our methods to understand our work, and that this is more than what is typically conveyed in similar studies. To address this concern, we have limited our discussion of the MAVE target sequence alignment and reference sequence selection components of our work to the recommended two paragraphs in the manuscript methods, and have moved additional detail to the manuscript supplementary material. The results section has been rewritten to primarily focus on the characterization of the resultant dataset and de-emphasize the methods we used to generate it.

>> MaveDB variants have the structure <proteinSequenceId>, <position>, <original amino

>> acid>, <new amino acid>.

> This is factually incorrect. The relevant excerpts about the structure of MaveDB variants

> are described in the paper introduction:

> For a given entry in MaveDB, a MAVE dataset describes sequence changes in MAVEHGVS(15), a format similar to the Human Genome Variation

> Society (HGVS) variant

> Specifically, while variants in HGVS are described in relation to accepted reference

> sequences (e.g. NP_003997.1:p.Trp24Cys), the vast majority of variants in MAVE score

> sets in MaveDB are described only in the context of the assayed sequence.

First you write that what I wrote is factually incorrect, then you write basically the same thing: that your changes are defined on a protein sequence. That's what I wrote. You seem that think that all protein sequences in databases match the genome. This is the misunderstanding here. But they don't, not always, and a lot of software already exists to solve this problem, map protein sequences that differ from the genome to the genome.

We invite the reviewer to carefully review the quoted text from our prior response, which was describing a factually incorrect characterization by the reviewer of the variant representation format used by the MaveDB resource. We also remind the reviewer that (as described throughout the manuscript text and in Figure 3A) while many MAVE datasets represent experimental data on protein sequences, 45% of scoresets use DNA sequences instead.

> Also, we must again respectfully point out to this reviewer that our work was a necessary

> advancement for making these data accessible to the community, as evidenced by the

> many integrations discussed in the manuscript.

I totally agree that this work is important, I wrote that myself in the very first sentence of my critique.

We thank the reviewer for this clarification that they find this work an important advance for the community.

What I criticize is the technical difficulty and the way this is implemented. You could have used just mapped from your protein to the Refseq sequence through an alignment, then use mutalyzer's position converter to go to the genome and be done with it. After having checked, I agree, not with R, you could have not used "proteinToGenome()" in Bioconductor, as that is not using an alignment (my fault). But in your paper, there is a lot of text, a special python package, various APIs, a UTA, and more dependencies. I have never heard of Gene Normalizer before, it looks like it could be a two column text file. All this overengineering will make the pipeline very difficult to run in the future or for someone else. Reading this paper, a student a bioinformatics could get the idea that the task target-protein-sequence + position -> genome is a hard problem.

We appreciate that the reviewer attempted to do a mapping themself to help us improve the efficiency of our approach. The issues the reviewer ran into when using the proteinToGenome method in Bioconductor is indicative of the general class of challenges we ran into for this study. Specifically, while many tools exist that can help address individual components of this study (and we have reused those tools where appropriate), there is no off-the-shelf tool that can “just work” across the highly heterogeneous nature of data submissions found in the MaveDB resource. We would like to further clarify that not all of the tools we used (e.g. the referenced Gene Normalizer) play a central role in a generalized mapping process, but they were helpful in addressing some specific challenges (e.g. processing gene metadata accompanying the MaveDB scoresets) that allowed us to address some of this submission heterogeneity.

We think that the reviewer will agree that with our substantial revisions to the depth of the manuscript methods and focus of the results, we have greatly reduced the amount of material the proverbial student reader would need to review to understand the process and impact of our work.

All data that is needed is the RefSeq alignment to the genome from NCBI as a GFF3 and a BLAST or BLAT alignment of your MaveDB protein or nucleotide sequences to RefSeq transcript annotated in MaveDB. All source code that is needed is a single Python loop to do the nucleotide or amino acid counting along these two alignments. I'm not sure how such a simple task is taking up pages of methods and dozens of acronyms.

We think this is an oversimplification of the needed tools to accomplish the efforts described in this study, and at odds with the reviewer’s earlier comments about the importance of our work and the challenges they experienced themself when attempting to implement the alternative solutions they had previously proposed. We reiterate that our study was not limited only to creating an alignment of the sequence in each scoreset, but also selecting a representative transcript (and corresponding protein sequence) from such alignments, re-normalization and validation of variants described on both reference and non-reference sequences, and standardized representation of variant provenance to enable contextual reuse of MAVE data in downstream resources. We also emphasize again that there is great heterogeneity in sequence and variant representation across scoresets submitted to MaveDB, requiring us to write many additional methods for data and metadata inference, validation, and standardization as described in the manuscript.

I agree that one problem if you want to use mutalyzer instead or transvar or hgvs, you still need to map from protein to RefSeq through an alignment, but that's not rocket science.

We appreciate the reviewer’s acknowledgement of the need for an alignment-based approach to a reference in conjunction with prior solutions for translating across protein, transcript, and genome contexts. This is, in essence, what we have already done for the alignment component of this study. We agree with the reviewer that this is not a conceptually challenging task, though it is procedurally difficult due to the complexity and heterogeneity of submitted datasets.

Overall, we appreciate the time and effort the reviewer has put into this second review of our manuscript, and their acknowledgement in the editorial system that we have satisfactorily responded to the comments from their initial review. We recognize the importance of creating a concise and impactful representation of our work, and this reviewer has helped us greatly improve the readability and approachability of our manuscript and associated code.
